# Dengue Virus Inhibits Immune Responses in *Aedes aegypti* Cells

**DOI:** 10.1371/journal.pone.0010678

**Published:** 2010-05-18

**Authors:** Shuzhen Sim, George Dimopoulos

**Affiliations:** W. Harry Feinstone Department of Molecular Microbiology and Immunology, Bloomberg School of Public Health, Johns Hopkins University, Baltimore, Maryland, United States of America; University Hospital Zurich, Switzerland

## Abstract

The ability of many viruses to manipulate the host antiviral immune response often results in complex host-pathogen interactions. In order to study the interaction of dengue virus (DENV) with the *Aedes aegypti* immune response, we have characterized the DENV infection-responsive transcriptome of the immune-competent *A. aegypti* cell line Aag2. As in mosquitoes, DENV infection transcriptionally activated the cell line Toll pathway and a variety of cellular physiological systems. Most notably, however, DENV infection down-regulated the expression levels of numerous immune signaling molecules and antimicrobial peptides (AMPs). Functional assays showed that transcriptional induction of AMPs from the Toll and IMD pathways in response to bacterial challenge is impaired in DENV-infected cells. In addition, *Escherichia coli*, a Gram-negative bacteria species, grew better when co-cultured with DENV-infected cells than with uninfected cells, suggesting a decreased production of AMPs from the IMD pathway in virus-infected cells. Pre-stimulation of the cell line with Gram-positive bacteria prior to DENV infection had no effect on DENV titers, while pre-stimulation with Gram-negative bacteria resulted in an increase in DENV titers. These results indicate that DENV is capable of actively suppressing immune responses in the cells it infects, a phenomenon that may have important consequences for virus transmission and insect physiology.

## Introduction

The incidence and geographic range of dengue and dengue hemorrhagic fever has increased dramatically in recent decades. With 2.5 billion people now living in areas at risk for epidemic transmission, dengue has become the most important mosquito-borne viral disease affecting humans [1]. Dengue virus (DENV) is a positive-strand RNA virus of the family *Flaviviridae*, genus *Flavivirus*. It exists as four closely related but antigenically distinct serotypes (DENV-1, -2, -3, and -4), all of which have *Aedes aegypti* mosquitoes as their primary vector, with *A. albopictus* as a secondary vector.

Mosquitoes, like all insects, are exposed to a variety of microbes in their natural habitats, and possess an innate immune system that is capable of mounting a potent response against microbial challenge. The insect innate immune response is largely regulated by three main immune signaling pathways: the Toll, immune deficiency (IMD) and Janus kinase signal transducer and activator of transcription (JAK-STAT) pathways. The Toll pathway is involved in defense against fungi, Gram-positive bacteria, and viruses [2]–[4], and has been found to be specifically involved in the *A. aegypti* anti-DENV response [5]. The IMD pathway has a major role in the production of antimicrobial peptides (AMPs) that control Gram-negative bacterial infections [6], and has more recently been shown to control Sindbis virus (SINV) infection in *Drosophila melanogaster* [7]. Likewise, the JAK-STAT pathway has been implicated in antiviral defense in insects [8], including defense against DENV in *A. aegypti* [9].

Despite the well-documented involvement of the Toll, IMD, and JAK-STAT pathways in insect antiviral defense, very little is known about how these pathways are activated by viruses at the molecular level. For example, viral pathogen-associated molecular patterns (PAMPs) and their associated insect pattern recognition receptors (PRRs) have not yet been identified, and only a few putative antiviral effector molecules have been identified [9], [10].

The host antiviral response is often countered by the ability of viruses to suppress or evade host immune responses. For example, several DENV non-structural proteins are known to play roles in the suppression of the mammalian interferon signaling pathway [11]–[13]. However, although this suppression and the mechanisms by which it occurs are well-characterized in the vertebrate system, very little is known about whether similar processes are at work in the mosquito vector.

In the mosquito, a detailed molecular characterization of the innate response to virus infection is complicated by the presence of many different tissues and body compartments. For this reason, we decided to characterize the mosquito anti-DENV response using the immune-competent Aag2 *Aedes aegypti* cell line [14], [15]. We reasoned that the cell line would be a more homogenous and sensitive system, thus allowing us to detect more subtle changes in gene expression in response to viral infection.

Our microarray analysis of the Aag2 DENV-responsive transcriptome indicated that DENV regulates a large number of genes from diverse classes in the Aag2 cell line, and, most strikingly, down-regulates a number of immune effectors and signaling molecules, suggesting that the virus is capable of inhibiting immune pathways in these cells. Functional assays indicated that DENV-infected cells are less capable of mounting an immune response against secondary bacterial challenge, and challenge with immune-response elicitors prior to DENV infection did not result in reduced virus infection, suggesting that the virus is actively suppressing immune pathways rather than failing to trigger them.

## Results

### a) Cell line transcriptome responses to DENV

**Figure 1:**
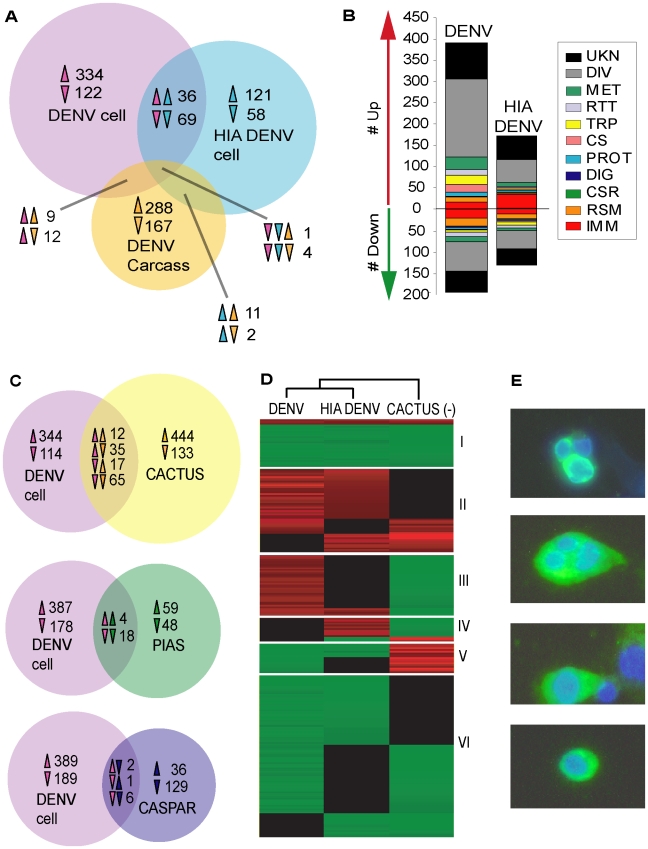
Transcriptional regulation of genes in the Aag2 cell line in response to live dengue virus (DENV) and heat-inactivated dengue virus (HIA DENV) infection. A. Venn diagram showing the numbers of unique and commonly regulated genes in DENV- and HIA DENV-infected cells and DENV-infected mosquito carcass. Arrows indicate the direction of gene regulation. B. Functional classification of significantly regulated genes in DENV and HIA DENV infection; arrows indicate the direction of gene regulation. Functional group abbreviations are as follows: UNK, unknown functions; DIV, diverse functions; MET, metabolism; RTT, replication, transcription, and translation; TRP, transport; CS, cytoskeletal and structural; PROT, proteolysis; DIG, blood and sugar food digestive; CSR, chemosensory reception; RSM, redox, stress and mitochondrion; IMM, immunity. C. Comparative analysis of the DENV infection-responsive cell line transcriptome and the Toll-, IMD-, and JAK-STAT pathway-regulated mosquito transcriptomes. Venn diagrams show the numbers of unique and commonly regulated genes in DENV-infected Aag2 cells and Cactus-, Caspar-, and PIAS-silenced *A. aegypti* mosquitoes. Arrows indicate the direction of gene regulation. D. Cluster analysis of 238 genes that were regulated in at least two of three treatments: DENV infection in the cell line, HIA DENV infection in the cell line, Cactus silencing in *A. aegypti* mosquitoes. All genes presented in this cluster analysis are listed in Table S6. E. Detection of DENV in Aag2 cells by indirect immunofluorescence assay using mouse hyperimmune ascitic fluid specific for DENV2 (CDC) and an AlexaFluor488-conjugated goat anti-mouse secondary antibody. Blue: DAPI, Green: FITC.

In accordance with previous studies [16], we found the Aag2 cell line readily permissible to infection with DENV (Figure 1E). In order to assess the global transcriptional response pattern of the Aag2 cell line to DENV infection, we employed a whole genome oligonucleotide microarray to compare transcript abundance in non-challenged cells to that in cells that had been challenged with either live virus (DENV) or heat-inactivated virus (HIA DENV) at an MOI of 1, at 48h post-infection (pi). This time point is relatively early in DENV infection, and was chosen to allow for sampling of the transcriptome while the virus was actively replicating: the length of one DENV replication cycle is estimated to be ∼30h [17], and a growth curve of DENV infection in Aag2 cells showed that DENV titers were increasing steadily at 48hpi, peaking only around 5 days pi (data not shown).

DENV infection significantly regulated 587 genes in the cell line (391 induced and 196 repressed), while HIA DENV exposure resulted in the regulation of 302 genes (170 up-regulated and 132 down-regulated) (Figure 1A), suggesting that virus replication accounts for a large proportion of the cellular response to DENV. A total of 36 genes were up-regulated and 74 were down-regulated by both challenges (Figure 1A), suggesting that these genes may be regulated in response to the recognition of viral PAMPs. Among the genes that were up-regulated by both challenges were several hypothetical proteins containing transmembrane domains (including one containing leucine-rich repeats [LRRs]), and a tyrosine-kinase related protein, which could be involved in virus recognition or downstream signaling processes. The fact that we did not find oppositely regulated genes under the two experimental conditions suggests that live and HIA DENV may trigger very similar cellular pathways (Figure 1A).

Live DENV infection significantly regulated genes that are involved in diverse cellular physiological systems (Figure 1B). Some 37 genes with putative immune functions were significantly regulated by DENV (Table S1). Of these, 16 were up-regulated, including the Toll pathway components Toll and Cactus, PRRs (a peptidoglycan recognition protein [PGRP] and a galactoside-binding lectin [GALE]), and signal modulators (two C-type lectins [CTLs] and a scavenger receptor). Interestingly, a putative heat-shock protein, HSP70, which has been implicated as part of the DENV receptor complex in human cells [18], was also among the up-regulated immune-related genes. In addition, 29 of the 41 significantly regulated genes with putative metabolic functions were up-regulated, perhaps indicating a shift in the metabolic state of the cell to support virus replication [19]. For example, several glucosyl/glucuronosyl transferases (enzymes involved in protein glycosylation) were induced, as was as Sec24B, a component of the COPII protein complex required for vesicle budding from the ER. This could indicate increased production and trafficking of viral proteins. There was also an up-regulation of enzymes involved in amino acid biosynthesis and fatty acid biosynthesis and elongation, perhaps indicating the increased use of host pathways for the synthesis of viral components. Of the 26 significantly regulated genes with putative transport functions, 21 were up-regulated, possibly as a result of increased vesicle transport or the use of transmembrane transporters as virus receptors. DENV also induced the transcription of a DEAD box-ATP-dependent RNA helicase and a DEAD box polypeptide-encoding gene; this finding is noteworthy because it was recently shown that *Drosophila* Dicer-2, a DExD/H-box helicase, is capable of sensing viral dsRNA and inducing the production of a putative antiviral effector molecule [10].

Six ubiquitin-proteasome pathway-related genes were induced in live DENV-infected cells. Transcriptional activation of this pathway has also been reported in DENV-infected mammalian cells [20], [21], and treatment with proteasome inhibitors has been reported to impair DENV replication in liver-derived HepG2 cells [20]. This suggests that the virus may use components of this pathway for replication in both invertebrate and vertebrate systems.

Unexpectedly, HIA DENV induced twice as many immune-related genes as did live DENV (33 of 170 up-regulated genes, or 19.4% for HIA DENV, as compared to 16 of 391, or 4.1% for DENV) (Figure 1B, Table S1). Transcriptional activation of Toll pathway components also occurred in HIA DENV-infected cells, with up-regulation of two Tolls, MyD88, and Rel1. A broader range of immune-related genes was up-regulated in HIA DENV-infected cells, including PRRs (a Gram-negative bacteria-binding protein [GNBP] and three fibrinogen and fibronectin related proteins [FREPs]), signal modulators (several CTLs, a clip-domain serine protease [CLIP] and a serine protease inhibitor [serpin]), and several oxidative defense enzymes. Other up-regulated immune genes included a transcript from the Down syndrome cell adhesion molecule (DSCAM) gene, which in *Anopheles gambiae* generates a wide range of PRRs that are involved in the defense against bacteria and *Plasmodium* [22], and two proteins with suppressor of cytokine signaling (SOCS) domains. Members of the SOCS gene family are induced upon JAK-STAT pathway activation and function in a negative feedback loop to inhibit the catalytic activity of JAK [23].

Cell line challenges led to the regulation of a unique set of genes when compared to DENV infection of the mosquito carcass [5] (Figure 1A). The transcript abundance of certain genes in the mosquito carcass could reflect an averaging of gene expression levels across a variety of tissues and organs and therefore result in a lack of difference between the compared samples, whereas the cell line is likely a more homogenous, simpler and more sensitive system.

The Toll, JAK-STAT, and IMD pathways are negatively regulated by Cactus, PIAS, and Caspar, respectively. Our group has previously characterized the transcriptomes of immune pathway-activated *A. aegypti* through RNAi-mediated depletion of these negative regulators [5], [9]. A comparison of the DENV-infected cell transcriptome with those of Cactus-, PIAS-, and Caspar-silenced mosquitoes revealed the greatest overlap with the Cactus-silenced (or Rel1-activated) transcriptome (22% of the DENV-infected cell transcriptome), followed by the PIAS (3.7%) and Caspar (1.5%) silencing-regulated transcriptomes (Figure 1C). Interestingly, four AMPs (three cecropins and one defensin) were down-regulated in the virus-challenged cell line as well as in JAK-STAT pathway activated (by PIAS silencing) mosquitoes, suggesting that JAK-STAT pathway activation during DENV infection of the cell line may be responsible for the regulation of these immune effectors. These findings are in agreement with our previous observation that the Toll and JAK-STAT pathways regulate anti-DENV defense in adult mosquitoes, but the IMD pathway does not [5], [9].

Strikingly and unexpectedly, DENV infection significantly down-regulated a large number of immune-related genes (21 of 196, or 10.7% of all down-regulated genes) in the cell line. Among the repressed immune genes were PRRs (a PGRP, a GNBP, two FREPs, and a thio-ester containing protein [TEP]), signal modulators (several serine proteases and CTLs) and AMPs (six cecropins and two defensins) (Table S1).

In live mosquitoes, RNAi-mediated depletion of the Toll pathway negative regulator Cactus results in the induction of numerous immunity-related genes [5]. To further explore the repression of immune genes observed during DENV infection of the cell line, we performed a cluster analysis of significantly regulated genes in challenged cells and in Cactus-silenced (or Rel1-activated) mosquitoes. This analysis revealed that numerous genes were oppositely regulated in DENV-infected cells and Cactus-silenced mosquitoes (expression clusters III and V, Figure 1D). Of particular interest was expression cluster V, which was enriched in immune genes that were repressed by DENV infection in the cell line but induced by Cactus silencing in mosquitoes.

The simultaneous up- and down-regulation of immune-related genes in the cell line by DENV is intriguing and suggests that DENV selectively evades or suppresses certain aspects of the mosquito's immune response. Analysis of the infection-responsive genes did not allow us to narrow this transcription signature down to a specific pathway or function, since many of the repressed genes (such as AMPs) can be regulated by one or more pathways.

### b) DENV-infected cells are compromised in mounting an immune response to secondary bacterial challenge

**Figure 2:**
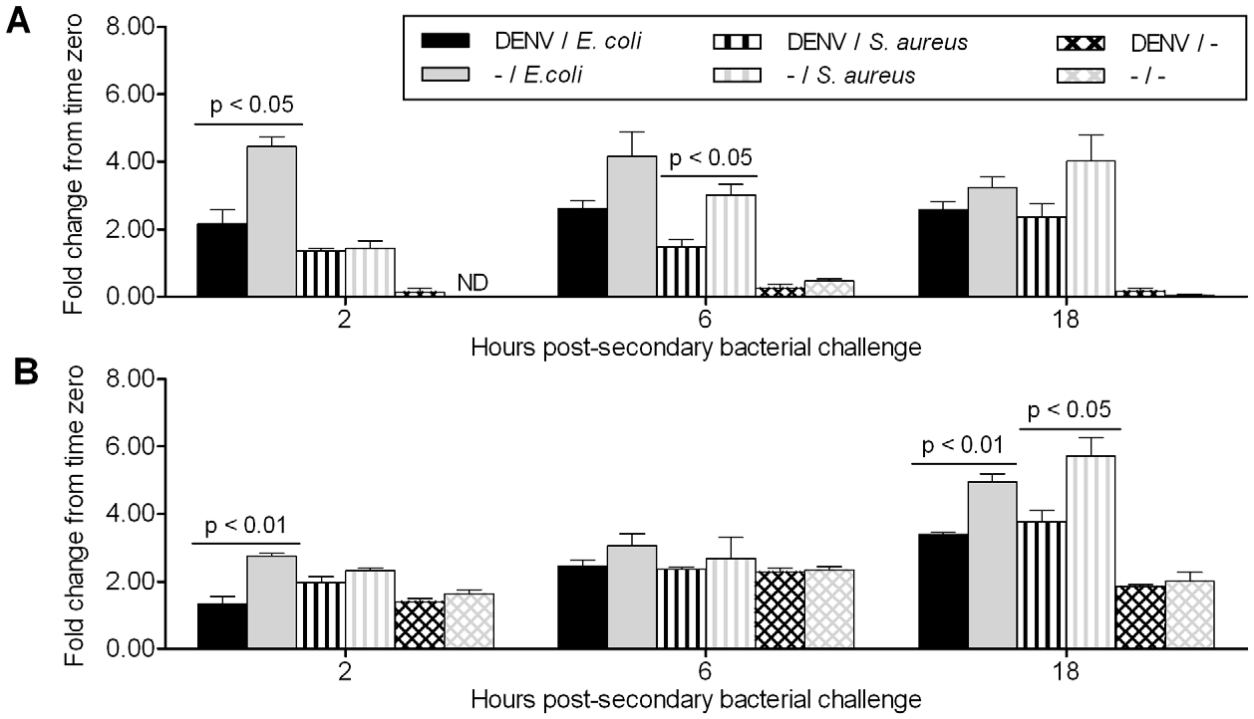
DENV-infected cells are less able to produce antimicrobial peptides in response to secondary bacterial challenge. Aag2 cells were DENV- or mock-infected, then challenged or mock-challenged with heat-killed *E. coli* or *S. aureus*. The bar charts show the -fold change in (A) cecropin and (B) defensin gene expression levels relative to levels at the 0-h time point for each sample, as measured by semi-quantitative PCR. Error bars indicate the standard error of the mean; ND, non-detectable.

Challenge of mosquito cells with bacteria typically results in a robust induction of AMP expression in the cells. We hypothesized that if DENV suppresses immune signal transduction pathways and thereby transcription of effectors in the cell line, DENV-infected cells may be less able to mount an immune response to a secondary challenge with bacteria. Therefore, we infected cells with DENV at an MOI of 1 for 48 h, and then challenged them with 10 MOI of heat-killed *E. coli* (a Gram-negative bacteria species, known to activate the IMD pathway) or *S. aureus* (Gram-positive, known to activate the Toll pathway). Cell lysates were harvested at 2, 6, and 18 h after bacterial challenge, and expression levels of the AMP genes cecropin and defensin were measured by semi-quantitative PCR (Figure 2).

Secondary challenge with either bacterial species resulted in the rapid and robust induction of cecropin expression when compared to the unchallenged controls in the case of both DENV- and control mock-infected cells at all time points (p<0.01) (Figure 2). However, cecropin induction levels were lower in DENV-infected cells: At 2 h post-*E. coli* challenge, cecropin levels in mock-infected cells were >2-fold higher than in DENV-infected cells (p<0.05), and the same was true at 6 h post-*S.aureus* challenge (p<0.05) (Figure 2A). The difference in the kinetics of cecropin induction by the two bacterial species may potentially be explained by inherent differences in how Gram-negative and Gram-positive bacteria trigger an immune response.

As was the case for cecropin expression, significantly lower levels of defensin were produced by DENV-infected cells than by mock-infected cells at 2 h post-*E. coli* stimulation (p<0.01). In fact, while *E. coli-*challenged mock-infected cells induced significantly higher levels of defensin than did unchallenged cells (p<0.05), defensin levels for *E. coli-*challenged DENV-infected cells were not significantly different from those of unchallenged cells (Figure 2B). In addition, DENV-infected cells also produced significantly lower defensin levels at 18 hours post-challenge with both *E. coli* (p<0.01) and *S. aureus* (p<0.05) (Figure 2B).

At 6 h after *E. coli* and *S. aureus* challenge, the level of defensin expression in bacteria-challenged cells was not significantly different from that in non-challenged cells (Figure 2B). This result reflects the fact that the unchallenged cells displayed slightly higher levels of defensin at this time point, and may also reflect the natural variation of baseline defensin levels in this cell line over time.

These data suggest that DENV is capable of repressing the induction of AMPs that are regulated by both the Toll and IMD pathways.

### c) DENV-infected cells are compromised in inhibiting the growth of bacteria in co-culture

A variety of insect cells display antibacterial activities and can therefore inhibit the growth of bacteria in co-culture, presumably through phagocytosis and the secretion of AMPs into the cell culture medium [24]. Since the data presented above suggested that DENV is able to repress AMP production in response to bacterial challenge, we hypothesized that prior infection with DENV would result in a reduced antibacterial activity. In order to test this hypothesis, cells were infected with DENV or mock-infected for 48 h, and then challenged with live bacteria in 10-fold dilutions ranging from 1.4×10^7^ to 1.4 bacterial cells per well. The OD595 was determined at 12 h after bacterial challenge as a measure of bacterial growth.

**Figure 3:**
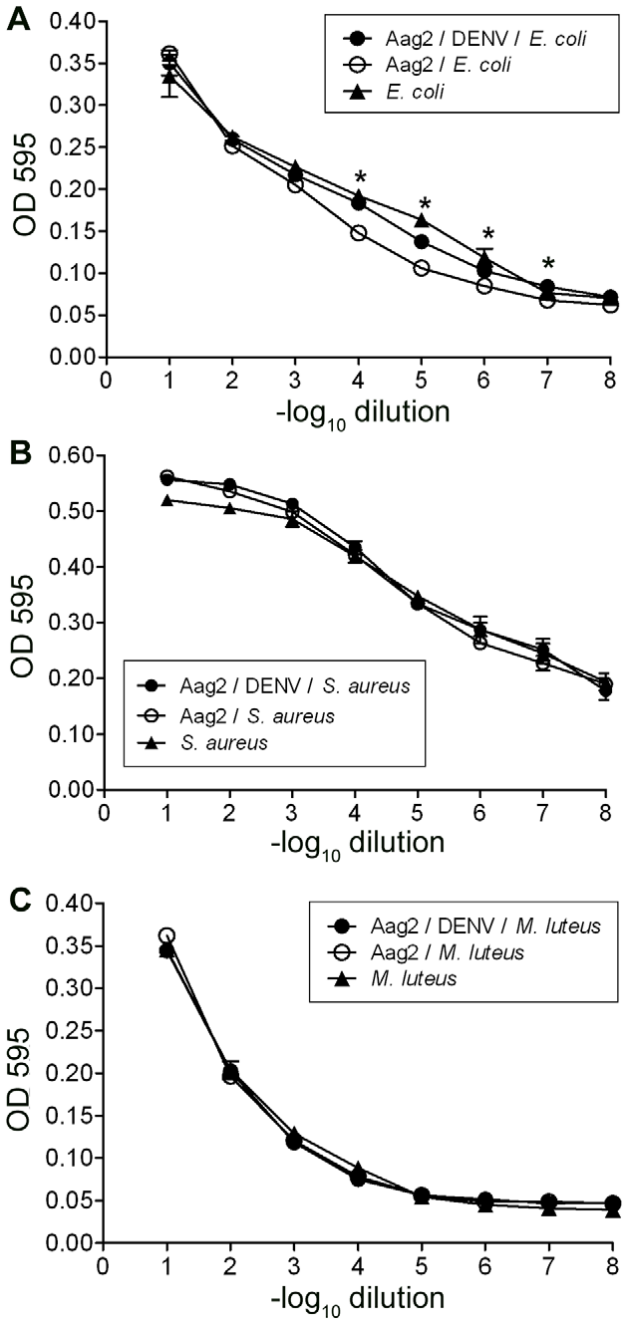
DENV-infected cells are less able to inhibit the growth of Gram-negative bacteria in co-culture. Eight 10-fold dilutions of Gram-positive or Gram-negative bacteria were inoculated into the cell culture medium in 96-well plates containing DENV- or mock-infected Aag2 cells, or cell culture medium alone. The graphs show OD595 as a measure of bacterial growth after a 12-h incubation at 28°C for (A) *E. coli*, (B) *S. aureus*, and (C) *M. luteus*. * represents p<0.05 in a Student's t-test comparing OD595 of bacteria incubated with DENV- and mock-infected cells. Error bars indicate the standard error of the mean; note that in many cases the error bars are obscured by the data point.

As expected, *E.coli* co-cultured with Aag2 cells grew to a lower OD595 than *E. coli* grown alone (p<0.05 at dilutions 3 to 6, dilution 8) (Figure 3A). In addition, *E. coli* co-cultured with DENV-infected Aag2 cells grew to a higher OD595 than *E. coli* co-cultured with uninfected cells (p<0.05 at dilutions 4 to 7) (Figure 3A), in agreement with our hypothesis that DENV infection compromises the ability of the cells to mount an immune response against bacteria. However, co-culture with uninfected or DENV-infected cells did not affect the OD595 of the Gram-positive bacteria *S. aureus* and *M. luteus* (Figure 3B and C), suggesting that these species are more resistant to the AMPs produced by the cells. In a separate experiment, we incubated DENV and bacteria together for 12 h and confirmed that the virus does not have a direct effect on OD595 (data not shown).

### d) Prior immune pathway stimulation in the cell line fails to suppress DENV infection

The lack of immune gene up-regulation by DENV infection that we observed could be due to the virus evading or failing to trigger an immune response, or alternatively to an active immune suppression mechanism. Therefore, we wanted to determine whether pre-immune activation by challenging with Gram-positive or Gram-negative bacteria prior to virus infection would affect DENV titers derived from the cells. For this purpose, cells were stimulated with 10 MOI of heat-killed *E.coli* or *S. aureus* for 24 h prior to infection with DENV at an MOI of 0.01, and DENV titers were determined every 24 h thereafter.

**Figure 4:**
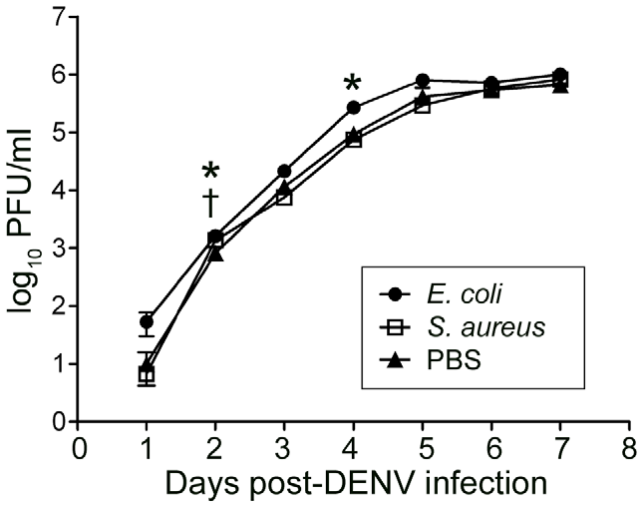
Effect of pre-immune stimulation on DENV titers in the cell line. Aag2 cells were pre-immune stimulated by the addition of heat-killed *E. coli* and *S. aureus* 24 h prior to DENV infection. The graph shows log_10_ DENV titers over 7 days as determined by plaque assay for cells pre-stimulated with heat-killed *E. coli* or *S. aureus*, or mock-stimulated with PBS. † represents p<0.05 in a Student's t-test comparing DENV titers in *S. aureus-* and mock-stimulated cells; * represents p<0.05 in a Student's t-test comparing DENV titers in *E. coli-* and mock-stimulated cells. Error bars indicate the standard error of the mean.

DENV titers in cells pre-challenged with *S. aureus* closely paralleled those in control cells (pre-treated with PBS) except at 2 days post-DENV infection, when *S. aureus-*treated cells produced significantly higher DENV titers than control cells (p<0.01) (Figure 4). This suggests that DENV is actively suppressing at least part of the immune response that is triggered by exposure to *S. aureus*, or alternatively that the virus is not affected by this response. Another explanation could be that *S. aureus* triggers other non-immune cellular pathways that favorably affect virus growth.

Unexpectedly, pre-challenge of the cells with *E. coli* resulted in higher DENV titers at all time points post-virus infection, significantly at 2 and 4 days post-DENV infection as compared to the PBS-treated controls (p<0.01 and p<0.05 respectively) (Figure 4). A plausible explanation for these results is that the *E. coli* challenge-elicited immune response exhausted the cell's capacity to produce anti-dengue effectors. This situation would imply that in a normal infection scenario, the IMD pathway also participates in anti-DENV defense, together with the Toll pathway.

## Discussion

In order to examine the response of *A. aegypti* to DENV infection in a more homogenous and sensitive system, we have characterized the DENV infection-responsive transcriptome of the immune-competent *A. aegypti* cell line Aag2. The cellular response to DENV infection involved a variety of physiological systems and functional gene classes, indicating that virus replication has a substantial effect on cell physiology.

DENV infection of the cell line and the mosquito carcass [5] resulted in the regulation of unique subsets of genes. These differences could be due to several factors: First, different time points were sampled in the two data sets: The cell line transcriptome was determined at 48 h post-infection, whereas the mosquito transcriptome was characterized at 10 days post-infection, which is relatively late in the infection process. It would be interesting to carry out a microarray analysis of DENV infection in the mosquito at the very early stages of infection (eg<24 h post-infectious bloodmeal) and compare the results to our cell line data. Second, as mentioned above, the mosquito carcass comprises many different tissue types and organs; thus, the transcriptome may reflect an averaging across these heterogeneities as well as the sum total of the transcript abundance in the different tissues and cell types. Despite these differences, however, viral challenge of the cell line also transcriptionally activated several Toll pathway components, further strengthening the evidence for the role of this pathway in antiviral defense.

A striking difference between DENV infection in the cell line and in the mosquito carcass was the relative lack of immune gene activation in the cell line. Immune genes made up only 4.1% of up-regulated genes in the cell line but constituted 22.5% of up-regulated genes in the mosquito carcass. Further, DENV infection also resulted in a broad down-regulation of immune genes in the cell line. This suggests that the immune modulation observed in DENV-infected cells is an active suppression mechanism that may require a replicating virus or, alternatively, could be triggered by the interaction with, or internalization of, the virus in absence of replication. Immune genes induced by exposure to HIA DENV comprised 19.4% of up-regulated genes, a value comparable to that for the mosquito carcass, and a broad down-regulation of immune genes was not observed.

We have further demonstrated that DENV-infected cells are less able to produce transcripts of the AMPs cecropin and defensin in response to challenge with Gram-positive and Gram–negative bacteria, suggesting that DENV is repressing the immune pathways that mediate these responses. In addition, *E. coli* co-cultured with DENV-infected cells was able to grow to a higher OD than bacteria cultured with uninfected cells, providing further evidence that DENV suppresses an antibacterial immune response.

Since cellular metabolism is presumably altered by DENV infection, we cannot rule out the possibility that the increased proliferation of *E. coli* in co-culture with DENV-infected cells could be attributed to changes in levels of metabolites released from virus-infected cells, instead of to decreased AMP levels. For example, several enzymes involved in fatty acid and amino acid biosynthesis were induced in DENV-infected cells. However, given the nutrient-rich nature of cell culture media, we find it unlikely that changes in metabolite levels released from the cells would have a marked effect on bacterial growth. Taking into account the finding that DENV-infected cells are impaired in their ability to produce AMPs in response to secondary bacterial challenge, it is likely that the increased bacterial proliferation is at least partly due to decreased AMP levels.

Further, while it cannot be ruled out that the virus is causing an overall down-regulation of host gene expression instead of specifically targeting immune signaling pathways, our microarray data do not suggest this: DENV induced twice the number of genes it repressed, and immune-related genes were the largest specific class of down-regulated genes (excluding those with unknown and diverse functions).

Many viruses suppress or evade immune signaling pathways, and DENV is no exception. The DENV NS4B protein antagonizes the vertebrate IFN pathway by blocking STAT1 phosphorylation and activation, preventing STAT dimers from translocating to the nucleus [11], [12], and DENV NS5 has more recently been shown to bind STAT2 and target it for proteasomal degradation [13]. The related flavivirus Japanese encephalitis virus (JEV) inhibits STAT phosphorylation in both vertebrate [25], [26] and mosquito (C6/36) cells [27]. Semliki Forest virus (SFV) has been found to reduce Toll, IMD and JAK-STAT signaling in mosquito cells [22], and Sindbis virus (SINV) infection of *A. aegypti* mosquitoes has been suggested to inhibit the Toll pathway after an initial activation stage [28]. Insect viruses are also capable of immune pathway suppression: For example, the *Microplitis demolitor* bracovirus inhibits Toll and IMD signaling as well as antimicrobial melanization reactions [29]–[31].

In our study, immune challenge with Gram-positive bacteria (which have been shown to activate the Toll pathway) did not affect DENV titers, in agreement with what has been observed for SINV [22], and suggesting that DENV may actively suppress the Toll pathway rather than evade it. Prior immune challenge with Gram-negative bacteria, which are known to activate the IMD pathway, unexpectedly resulted in higher DENV titers. We speculate that robust stimulation of this pathway by *E. coli* challenge may have exhausted the cell's capacity to produce IMD pathway-regulated effectors, thus allowing DENV to replicate more freely. A similar effect was seen in *A. aegypti* mosquitoes, in which IMD pathway activation through Caspar silencing resulted in increased midgut DENV titers, although this increase was not statistically significant [5]. This interpretation would suggest that the IMD pathway plays a role in keeping DENV replication in check in a normal infection scenario. While up-regulation of IMD pathway components in response to DENV infection was not observed in the cell line or in the mosquito [5], this lack of regulation may be attributable to a balance between IMD pathway activation and DENV suppression of this pathway. It would be interesting to pursue the role of the IMD pathway in anti-DENV response, especially in light of two recent studies that have implicated this pathway in the antiviral response against SINV and cricket paralysis virus (CrPV) in *D. melanogaster* infection models [7], [32].

Our group has recently shown that there is also a down-regulation of several AMPs at early time points in DENV infection in live mosquitoes [33], suggesting that DENV may suppress immune responses at early infection stages before activating them at later time points, and indicating that our cell line data is applicable to a real-life infection scenario. An immune-suppressive effect of DENV in the mosquito at the early stages of infection could aid in establishing viral infection in the midgut. Since productively infected mosquitoes are capable of transmitting DENV for life, and also take multiple bloodmeals over a single gonatrophic cycle [34], an increased ability of DENV to overcome bottlenecks at the midgut infection stage could have implications for virus transmission. It would be interesting to determine whether DENV strains that differ in their ability to establish productive infections in mosquitoes also differ in their immune-suppressive activity.

In addition, a reduction in AMP expression in response to DENV infection may have physiological repercussions for the mosquito in nature. Mosquitoes are constantly exposed to bacteria and fungi in their environments, and rely on their innate immune system to keep bacterial infection in check. Suppression of immune pathways by arbovirus infection could lead to a proliferation of pathogenic or opportunistic bacteria, especially if viral infection brings about tissue damage that could increase the mosquito's susceptibility to septic infection.

There is growing interest in the similarities between DENV infection in mosquito and mammalian systems. A recent study found that numerous host factors required for DENV replication are conserved between invertebrate and vertebrate hosts [35]. Our microarray gene expression data revealed some overlaps between the mosquito and mammalian cell DENV-regulated transcriptomes, including up-regulation of ubiquitin-proteasome pathway components [20], [21], as well as cytoskeletal- and transport-related genes [20]. These overlaps suggest that drug targets developed for use in humans may also be useful for interventions in the vector.

In summary, the data presented here indicate that DENV is capable of inhibiting immune pathway activation in mosquito cell lines. Because of its small genome size and the high degree of conservation between the invertebrate and vertebrate innate immune systems, it is intriguing to hypothesize that DENV has evolved similar methods of immune suppression in both mosquitoes and humans. Our future studies will focus on the mechanisms by which DENV causes immune suppression in the invertebrate, as well as the effect that this suppression has on virus transmission.

## Materials and Methods

### a) Cell culture and DENV infection

*Aedes aegypti* Aag2 cells [14], [15] were maintained at 28°C in Schneider's *Drosophila* media with L-glutamine supplemented with 10% FBS and 1% penicillin/streptomycin, and were passaged at a 1∶10 dilution every 4–5 days. At 70–80% confluency, the cells reached a density of ∼1.8×10^5^ cells/cm^2^.

For infection, Aag2 cells were seeded in 6-, 24-, or 96-well plates to a confluency of 80%. DENV2 New Guinea C (NGC) diluted to the desired multiplicity of infection (MOI) in infection medium (Schneider's *Drosophila* medium with L-glutamine, supplemented with 2% FBS, 1% penicillin/streptomycin, and 1% non-essential amino acids) was then added to the cells. After rocking at room temperature for 1 h, the inoculum was aspirated, and the appropriate volume of infection medium was added to top up each well of the plate. Plates were incubated at 28°C for the duration of the experiment.

### b) Indirect immunofluorescence assay

To confirm infection, Aag2 cells infected with DENV2 an MOI of 1.0 were fixed in acetone on a microscope slide for 10 min at −20°C. After blocking for 1 h at room temperature in 10% goat serum, 0.1% TritonX-100 and 0.2% BSA in PBS, the slide was incubated with DENV2 mouse hyperimmune ascitic fluid (MHIAF, specific for DENV-2, CDC), followed by Alexa-Fluor 488-conjugated goat anti-mouse IgG for 1 hour each at room temperature. Slides were mounted with ProLong Gold anti-fade reagent with DAPI, and visualized under a fluorescent microscope.

### c) Microarray gene expression analysis

Aag2 cells were seeded to a confluency of 80% in 6-well plates and infected in triplicate with the following:

- DENV at an MOI of 1.
- Heat-inactivated DENV (HIA DENV) at an MOI of 1. DENV was heat-inactivated by heating at 70°C for 1 h.
- PBS (mock-infected control).

After incubation at 28°C for 48 h, infected and control cells were lysed by the addition of 600 µl of Buffer RLT (Qiagen) and homogenized for 30s with a rotor-stator homogenizer. RNA was then extracted with the Qiagen RNeasy Mini Kit.

Two micrograms of total RNA were used for probe synthesis of cy3- and cy5-labeled cRNA, and hybridizations were carried out on an Agilent-based microarray platform. Hybridization intensities were determined with an Axon GenePix 4200AL scanner, and images were analyzed with Gene Pix software.

Expression data were processed and analyzed as previously described [5], [9]; in brief, background-subtracted median fluorescent vales were normalized with the LOWESS normalization method, and Cy5/Cy3 ratios from replicate assays were subjected to t-tests at a significance level of p<0.05 using TIGR MIDAS and MeV software. Expression data from replicate assays were averaged with the GEPAS microarray preprocessing software and logarithm (base 2)-transformed. Self–self-hybridizations have been used to determine the cutoff value for the significance of gene regulation on these types of microarrays to 0.78 in log2 scale, which corresponds to 1.71-fold regulation [36]. For genes with p<0.05, the average ratio was used as the final -fold change; for genes with p>0.05, the inconsistent replicates (with distance to the median of replicate ratios >0.8) were removed, and only the values from genes with at least two replicates in the same direction of regulation were further averaged. Numeric microarray gene expression data are presented in Table S2. We have made all microarray data MIAME compliant and available through the public databases of NCBI-GEO (accession numbers: GSM472993, GSM472992, GSM472991, GSM472990, GSM472989, GSM472988).

### d) DENV-bacteria co-infections

Aag2 cells seeded in 24-well plates were infected or mock-infected with DENV for 48 h at an MOI of 1 [15]. Heat-killed *Escherichia coli* or *Staphylococcus aureus* diluted in PBS was then added to the cell culture medium to an MOI of 10 (bacteria were heat-killed by heating at 70°C for 30 min). As a mock-challenged control, an equivalent volume of PBS was added to the cells. All conditions were performed in triplicate.

At 0, 2, 6 and 18 h after bacterial challenge, the media were aspirated, and the cells were lysed by the addition of 350 µl of Buffer RLT. Lysates were homogenized for 30 s with a rotor-stator homogenizer, and RNA was then extracted with the Qiagen RNeasy Mini Kit.

### e) Semi-quantitative PCR

RNA samples were DNAse-treated using the Ambion Turbo DNAse kit, and 1 µg of total RNA was then reverse-transcribed with Superscript III (Invitrogen) using oligo(dT) primers. Separate PCRs were carried out for each gene being analyzed, and products were separated by gel electrophoresis and visualized on a Fuji image documenter system. Signal intensities from gel bands were quantified with the ImageGauge software (Fuji), and each sample was normalized to transcript levels of the *A. aegypti* ribosomal S7 gene. The primer sequences used were:

Cecropin G (AAEL015515-RA):

F: 5′-TCACAAAGTTATTTCTCCTGATCG-3′

R: 5′-GCTTTAGCCCCAGCTACAAC-3′

Defensin C (AAEL003832-RA):

F: 5′-TTGTTTGCTTCGTTGCTCTTT-3′

R: 5′-ATCTCCTACACCGAACCCACT-3′

S7 (AAEL009496-RA):

F: 5′-GGGACAAATCGGCCAGGCTATC-3′

R: 5′-TCGTGGACGCTTCTGCTTGTTG-3′

Numeric data for the semi-quantitative PCR assays are presented in Table S3.

### f) Bacterial growth inhibition assays

Bacterial growth inhibition assays were carried out on the basis of the protocol described in [24]. Aag2 cells seeded in 96-well plates were infected or mock-infected with DENV at an MOI of 1, and 60 µl of antibiotic-free medium was added to each well after aspiration of the inoculums [15]. Wells containing 60 µl of medium alone were also included as a control. The cells were incubated at 28°C for 48 h to allow for the accumulation of antimicrobial effectors in the cell culture supernatant. Overnight cultures of *E. coli, S. aureus*, and *Micrococcus luteus* were washed in PBS and diluted to ∼1.0 OD600, and eight serial 10-fold dilutions of bacteria were prepared from the original dilution. Samples (140 µl) of each bacterial dilution were added to wells containing infected cells, mock-infected cells, or medium alone in the 96-well plates. Plates were incubated for 12 h at 28°C, and the OD595 was measured using a microplate reader (Molecular Devices). Each dilution and sample was replicated four times. Numeric OD595 values are presented in Table S4.

### g) Plaque assays

Aag2 cells seeded in 96-well plates were pre-challenged with 10 MOI of heat-killed *E. coli* and *S. aureus* or mock-challenged with PBS for 24 h prior to infection with DENV at an MOI of 0.01 [15]. Cell culture supernatants (200 µl) were harvested every 24 h up to 7 days and replaced with 200 µl of fresh infection medium.

DENV titers in harvested supernatants were determined by plaque assay. Samples were serially diluted and inoculated into C6/36 cells in 24-well plates. After a 5-day incubation period at 32°C and 5% CO_2_, plaque forming units (PFUs) were visualized by immunoperoxidase staining using mouse hyperimmune ascitic fluid (MHIAF, specific for DENV2, CDC) as the primary antibody and a goat anti-mouse horseradish peroxidase conjugate as the secondary antibody [5], [9]. Numeric DENV titers from the plaque assays are presented in Table S5.

## Supporting Information

Table S1 Significantly regulated putative immune- and infection-related genes in DENV- and HIA DENV-infected Aag2 cells, and their overlap with those in Cactus-silenced *A. aegypti* mosquitoes. Aag2 cells were harvested for microarray analysis at 48 h post-challenge with 1 MOI of DENV or HIA DENV. Mosquitoes were injected with dsRNA to Cactus at 2-4 days post-emergence, and samples were collected for microarray analysis at 4 days after injection.
(0.08 MB DOC)

Table S2 Significantly regulated genes in DENV- and HIA DENV-infected Aag2 cells. Functional group abbreviations: CS, cytoskeletal and structural; CSR, chemosensory reception; DIV, diverse functions; DIG, blood and sugar food digestive; IMM, immunity; MET, metabolism; PROT, proteolysis; RSM, redox, stress and mitochondrion; RTT, replication, transcription, and translation; TRP, transport; UNK, unknown functions.
(0.86 MB DOC)

Table S3 Averaged data from three biological replicate semi-quantitative PCR assays. Averaged data from three biological replicate semi-quantitative PCR assays of cecropin and defensin expression at varying time points post-secondary bacterial challenge of DENV- or mock-infected Aag2 cells. The fold change in gene expression compared to the 0 h time point is shown. p-values are for a Student's t-test comparing fold change in gene expression upon secondary bacterial challenge in DENV- and mock-infected cells. *, p<0.05; SEM, standard error of the mean; ND, non-detectable.
(0.05 MB DOC)

Table S4 Average of four biological replicate OD595 readings. Average of four biological replicate OD595 readings for (A) *E. coli*, (B) *S. aureus*, and (C) *M. luteus* after a 12-h incubation at 28°C with DENV- or mock-infected Aag2 cells. p-values are for a Student's t-test comparing OD595 of bacteria incubated with DENV- and mock-infected cells. *, p<0.05; SEM, standard error of the mean.
(0.06 MB DOC)

Table S5 Averaged data from three biological replicate plaque assays of DENV titers. Averaged data from three biological replicate plaque assays of DENV titers in cell culture supernatants following pre-immune stimulation with *E. coli*, *S. aureus*, or PBS. p-values are for a Student's t-test comparing DENV titers in bacteria-stimulated cells with PBS-treated cells. *, p<0.05; SEM, standard error of the mean.
(0.04 MB DOC)

Table S6 Cluster analysis. Cluster analysis of 238 genes that were regulated in at least two of three treatments: DENV infection in the cell line, HIA DENV infection in the cell line, Cactus silencing in *A. aegypti* mosquitoes (Figure 1D).
(0.36 MB DOC)
