# Supplementary material for: Dengue Virus Inhibits Immune Responses in Aedes aegypti Cells
Source: PLoS One. 2010 May 18;5(5):e10678. doi: 10.1371/journal.pone.0010678 (PMC2872661; doi:10.1371/journal.pone.0010678)
Supplement: Table S1 — Significantly regulated putative immune- and infection-related genes in DENV- and HIA DENV-infected Aag2 cells, and their overlap with those in Cactus-silenced A. aegypti mosquitoes. Aag2 cells were harvested for microarray analysis at 48 h post-challenge with 1 MOI of DENV or HIA DENV. Mosquitoes were injected with dsRNA to Cactus at 2-4 days post-emergence, and samples were collected for microarray analysis at 4 days after injection. (0.08 MB DOC) [file pone.0010678.s001.doc]

Table S1: Significantly regulated putative immune- and infection-related genes in DENV- and HIA DENV-infected Aag2 cells, and their overlap with those in Cactus-silenced *A. aegypti* mosquitoes. Aag2 cells were harvested for microarray analysis at 48 h post-challenge with 1 MOI of DENV or HIA DENV. Mosquitoes were injected with dsRNA to Cactus at 2-4 days post-emergence, and samples were collected for microarray analysis at 4 days after injection.

| **Gene ID** | **Name** | **Functional Group** |  | **Log_2_ fold** |  |
| --- | --- | --- | --- | --- | --- |
|  |  |  | **DENV** | **HIA DENV** | **CAC** |
| Aaeg:N49982 | CLIPB16 | Signal modulation | 0.778 | 1.526 |  |
| AAEL010951 | glutamate decarboxylase | Melanization | 0.814 | 0.894 |  |
| AAEL007363 | leucine rich transmembrane protein | Pattern recognition receptor | 1.055 | 0.837 | 0.993 |
| AAEL007613 | TOLL1A | Toll pathway | 1.179 |  | 1.307 |
| Aaeg:N51900 | HSC70-3 | Virus receptor | 0.789 |  | 0.964 |
| AAEL002166 | leucine rich repeat interacting protein | Pattern recognition receptor | 1.131 |  | -1.294 |
| AAEL000709 | CACT | Toll pathway | 0.929 |  |  |
| AAEL003119 | CTL6 | Signal modulation | 0.879 |  |  |
| AAEL014356 | CTLSE2 | Signal modulation | 0.843 |  |  |
| AAEL001914 | SCRAC1 | Signal modulation | 0.78 |  |  |
| Aaeg:N42080 | DDC | Melanization | 0.867 |  |  |
| AAEL002206 | GALE | Pattern recognition receptor | 1.043 |  |  |
| AAEL014989 | PGRPLD | Pattern recognition receptor | 0.855 |  |  |
| Aaeg:N41501 | CAT1A | Oxidative defense | 1.101 |  |  |
| AAEL005832 | programmed cell death | Apoptosis | 0.969 |  |  |
| AAEL003554 | leucine rich repeat protein | Pattern recognition receptor | 0.805 |  |  |
| AAEL011455 | CTLMA12 | Signal modulation |  | 1.622 | 2.473 |
| Aaeg:N18089 | DCE | Melanization |  | 1.409 | 1.366 |
| Aaeg:N32065 | CTL | Signal modulation |  | 1.315 | 1.197 |
| AAEL011619 | CTLGA8 | Signal modulation |  | 1.238 | 1.129 |
| AAEL007696 | REL1A | Toll pathway |  | 0.807 | 1.005 |
| AAEL006699 | FREP34 | Pattern recognition receptor |  | 1.546 | -1.297 |
| AAEL002704 | SRPN23 | Signal modulation |  | 1.149 | -0.917 |
| AAEL011764 | PPO10 | Oxidative defense |  | 1.537 | -0.844 |
| AAEL013417 | FREP24 | Pattern recognition receptor |  | 1.376 | -0.837 |
| AAEL000227 | SCRB8 | Signal modulation |  | 0.803 | -0.835 |
| AAEL007768 | MYD88 | Toll pathway |  | 0.818 |  |
| Aaeg:N35123 | HPX | Oxidative defense |  | 1.101 |  |
| Aaeg:N23036 | CLIP | Signal modulation |  | 1.242 |  |
| Aaeg:N52152 | SOCS | JAK-STAT pathway |  | 1.448 |  |
| Aaeg:N44241 | GPX | Oxidative defense |  | 0.857 |  |
| AAEL010992 | CTL8 | Signal modulation |  | 1.083 |  |
| AAEL008681 | CTL12 | Signal modulation |  | 1.048 |  |
| Aaeg:N12802 | WASP -like | Hematopoietic |  | 1.334 |  |
| AAEL009420 | SCRBQ1 | Signal modulation |  | 1.389 |  |
| Aaeg:N38609 | RANK1 | Ankyrin repeat |  | 1.044 |  |
| AAEL002354 | HPX5 | Oxidative defense |  | 1.271 |  |
| AAEL006137 | SRPN19 | Signal modulation |  | 1.475 |  |
| Aaeg:N6593 | DSCAM | Pattern recognition receptor |  | 1.311 |  |
| AAEL009551 | TOLL11 | Toll pathway |  | 1.042 |  |
| Aaeg:N40789 | SOCS | Signal transduction |  | 0.897 |  |
| AAEL000633 | TOLL8 | Toll pathway |  | 1.144 |  |
| Aaeg:N31575 | CTLMA15 | Signal modulation |  | 1.749 |  |
| AAEL000533 | CTL16 | Signal modulation |  | 1.314 |  |
| AAEL006702 | FREP33 | Pattern recognition receptor |  | 1.91 |  |
| AAEL009176 | GNBPB3 | Pattern recognition receptor |  | 0.832 |  |
| AAEL009384 | FREP5 | Pattern recognition receptor | -1.144 | -0.787 | 2.312 |
| AAEL015515 | CECG | Effector | -1.323 | -0.942 | 1.394 |
| AAEL000611 | CECE | Effector | -1.547 | -1.065 | 1.33 |
| AAEL000598 | CECD | Effector | -1.176 | -1.144 | 0.953 |
| AAEL003832 | DEFC | Effector | -0.992 | -1.016 | 0.95 |
| AAEL009474 | PGRPS1 | Pattern recognition receptor | -0.992 | -0.93 | 0.94 |
| AAEL001163 | TEP23 | Pattern recognition receptor | -1.076 | -1.001 | 0.81 |
| AAEL011009 | FREP13 | Pattern recognition receptor | -1.096 | -0.899 | -1.081 |
| Aaeg:N44512 | DCE | Melanization | -2.526 | -1.864 | -1.078 |
| AAEL005800 | CLIPE11 | Signal modulation | -1.037 |  | 2.639 |
| AAEL003632 | CLIPB39 | Signal modulation | -0.874 |  | 2.032 |
| AAEL011607 | CTLMA14 | Signal modulation | -0.848 |  | 1.649 |
| AAEL007107 | serine protease, putative | Signal modulation | -1.027 |  | 1.586 |
| AAEL002601 | CLIPA1 | Signal modulation | -0.907 |  | 1.258 |
| AAEL003857 | DEFD | Effector | -0.777 |  | 0.999 |
| AAEL007626 | GNBPA1 | Pattern recognition receptor | -0.906 |  | 0.953 |
| AAEL014251 | IAP5 | Apoptosis | -0.837 |  | -1.655 |
| AAEL000625 | CECF | Effector | -1.048 |  |  |
| Aaeg:N31904 | CTLMA14 | Signal modulation | -0.877 |  |  |
| AAEL000621 | CECN | Effector | -0.828 |  |  |
| AAEL006161 | CLIPB31 | Signal modulation | -0.81 |  |  |
| AAEL009842 | GALE12 | Pattern recognition receptor |  | -0.957 | 2.398 |
| AAEL000627 | CECA | Effector |  | -1.19 | 1.239 |
| AAEL004868 | hemomucin | Pattern recognition receptor |  | -0.811 | -0.807 |
