# Supplementary material for: Dengue Virus Inhibits Immune Responses in Aedes aegypti Cells
Source: PLoS One. 2010 May 18;5(5):e10678. doi: 10.1371/journal.pone.0010678 (PMC2872661; doi:10.1371/journal.pone.0010678)
Supplement: Table S2 — Significantly regulated genes in DENV- and HIA DENV-infected Aag2 cells. Functional group abbreviations: CS, cytoskeletal and structural; CSR, chemosensory reception; DIV, diverse functions; DIG, blood and sugar food digestive; IMM, immunity; MET, metabolism; PROT, proteolysis; RSM, redox, stress and mitochondrion; RTT, replication, transcription, and translation; TRP, transport; UNK, unknown functions. (0.86 MB DOC) [file pone.0010678.s002.doc]

**Table S2**: Significantly regulated genes in DENV- and HIA DENV-infected Aag2 cells. Functional group abbreviations: CS, cytoskeletal and structural; CSR, chemosensory reception; DIV, diverse functions; DIG, blood and sugar food digestive; IMM, immunity; MET, metabolism; PROT, proteolysis; RSM, redox, stress and mitochondrion; RTT, replication, transcription, and translation; TRP, transport; UNK, unknown functions.

|  |  |  |  | **Log_2_ fold** | |
| --- | --- | --- | --- | --- | --- |
| **Gene ID** | **Name** | **Immune name** | **Functional group** | **DENV** | **HIA DENV** |
| AAEL007898 | calmin |  | CS | 0.838 | 0.934 |
| AAEL001411 | myosin heavy chain, nonmuscle or smooth muscle |  | CS | 1.352 |  |
| AAEL013778 | f-actin capping protein alpha |  | CS | 1.209 |  |
| AAEL010510 | conserved hypothetical protein |  | CS | 1.133 |  |
| AAEL009733 | hypothetical protein |  | CS | 1.117 |  |
| AAEL011154 | hypothetical protein |  | CS | 1.09 |  |
| AAEL004936 | conserved hypothetical protein |  | CS | 1.084 |  |
| AAEL010979 | growth factor receptor-bound protein |  | CS | 1.045 |  |
| AAEL001477 | laminin alpha-1, 2 chain |  | CS | 1.016 |  |
| AAEL001904 | arp2/3 |  | CS | 0.947 |  |
| AAEL002771 | microtubule binding protein, putative |  | CS | 0.912 |  |
| AAEL005845 | beta chain spectrin |  | CS | 0.902 |  |
| AAEL013808 | fascin |  | CS | 0.874 |  |
| AAEL004440 | tubulin-specific chaperone e |  | CS | 0.87 |  |
| AAEL000700 | cadherin |  | CS | 0.859 |  |
| AAEL001574 | septin |  | CS | 0.819 |  |
| AAEL002761 | tropomyosin invertebrate |  | CS | 0.809 |  |
| AAEL004668 | septin |  | CS | 0.8 |  |
| AAEL000553 | tropomyosin, putative |  | CS | 0.796 |  |
| AAEL003027 | conserved hypothetical protein |  | CS |  | 1.137 |
| AAEL002185 | cuticle protein, putative |  | CS |  | 0.992 |
| AAEL009527 | conserved hypothetical protein |  | CS |  | 0.882 |
| AAEL006179 | tubulin alpha chain |  | CS | -1.537 | -1.116 |
| AAEL014483 | conserved hypothetical protein |  | CS | -0.85 |  |
| AAEL006340 | conserved hypothetical protein |  | CS |  | -1.035 |
| AAEL012207 | myosin light chain 1, putative |  | CS |  | -0.975 |
| AAEL008185 | conserved hypothetical protein |  | CSR | 0.851 |  |
| AAEL000048 | gustatory receptor for trehalose (trehalose receptor) |  | CSR |  | 3.172 |
| AAEL003593 | hypothetical protein |  | CSR |  | 1.295 |
| AAEL015071 | Gustatory receptor 64a, putative |  | CSR |  | -0.885 |
| AAEL013882 | tkr |  | DIV | 1.481 | 0.796 |
| AAEL004197 | hypothetical protein |  | DIV | 1.731 | 1.116 |
| AAEL007653 | allantoinase |  | DIV | 1.247 | 1.294 |
| AAEL000820 | dimethylaniline monooxygenase |  | DIV | 1.226 | 1.107 |
| AAEL014301 | hypothetical protein |  | DIV | 0.791 | 0.899 |
| AAEL003989 | GTP-binding protein alpha subunit, gna |  | DIV | 1.313 | 0.979 |
| AAEL011384 | hypothetical protein |  | DIV | 1.042 | 1.028 |
| AAEL010674 | hypothetical protein |  | DIV | 1.068 | 0.842 |
| AAEL007401 | roundabout, putative |  | DIV | 1.358 | 0.803 |
| AAEL006619 | conserved hypothetical protein |  | DIV | 1.643 | 0.896 |
| AAEL011105 | adducin |  | DIV | 0.864 | 0.797 |
| AAEL003220 | rho-type gtpase activating protein |  | DIV | 1.102 | 0.828 |
| AAEL013028 | zinc finger protein |  | DIV | 1.065 | 0.794 |
| AAEL010755 | hypothetical protein |  | DIV | 1.299 | 1.012 |
| AAEL011552 | hypothetical protein |  | DIV | 0.889 | 1.342 |
| AAEL010301 | conserved hypothetical protein |  | DIV | 0.821 | 0.81 |
| AAEL008027 | hypothetical protein |  | DIV | 1.227 | 0.789 |
| AAEL009377 | RAS protein, putative |  | DIV | 0.888 | 0.786 |
| AAEL014991 | hypothetical protein |  | DIV | 0.877 | 0.786 |
| AAEL004710 | spingomyelin synthetase |  | DIV | 1.71 |  |
| AAEL000405 | odd Oz protein |  | DIV | 1.675 |  |
| AAEL014746 | o-linked n-acetylglucosamine transferase, ogt |  | DIV | 1.629 |  |
| AAEL004715 | b-cell translocation protein |  | DIV | 1.534 |  |
| AAEL009646 | conserved hypothetical protein |  | DIV | 1.509 |  |
| AAEL003623 | conserved hypothetical protein |  | DIV | 1.476 |  |
| AAEL002899 | hypothetical protein |  | DIV | 1.473 |  |
| AAEL014042 | protein phosphatase pp2a regulatory subunit b |  | DIV | 1.45 |  |
| AAEL009249 | coronin |  | DIV | 1.428 |  |
| AAEL004351 | casein kinase |  | DIV | 1.418 |  |
| AAEL001441 | protein phosphatase 2c gamma |  | DIV | 1.417 |  |
| AAEL008806 | testis development protein prtd |  | DIV | 1.38 |  |
| AAEL004913 | adpbosylation factor, arf |  | DIV | 1.317 |  |
| AAEL003470 | conserved hypothetical protein |  | DIV | 1.314 |  |
| AAEL007966 | glutamate receptor, ionotropic ampa, subunit 1, 2, 3, putative |  | DIV | 1.312 |  |
| AAEL001434 | coronin |  | DIV | 1.296 |  |
| AAEL013969 | conserved hypothetical protein |  | DIV | 1.295 |  |
| AAEL012915 | als2cr7 |  | DIV | 1.257 |  |
| AAEL003571 | factor for adipocyte differentiation |  | DIV | 1.243 |  |
| AAEL013223 | hypothetical protein |  | DIV | 1.235 |  |
| AAEL001946 | four and a half lim domains |  | DIV | 1.234 |  |
| AAEL005795 | conserved hypothetical protein |  | DIV | 1.222 |  |
| AAEL007705 | hect E3 ubiquitin ligase |  | DIV | 1.222 |  |
| AAEL002705 | nucleolar protein c7b |  | DIV | 1.211 |  |
| AAEL005241 | lateral signaling target protein |  | DIV | 1.204 |  |
| AAEL001853 | rac-gtp binding protein |  | DIV | 1.204 |  |
| AAEL003698 | conserved hypothetical protein |  | DIV | 1.203 |  |
| AAEL009732 | rac gtpase |  | DIV | 1.187 |  |
| AAEL008879 | kynurenine 3-monooxygenase |  | DIV | 1.173 |  |
| AAEL004501 | s-adenosylmethionine synthetase |  | DIV | 1.166 |  |
| AAEL003145 | bestrophin 2,3,4 |  | DIV | 1.165 |  |
| AAEL006786 | gtpase_rho |  | DIV | 1.164 |  |
| AAEL008171 | double-stranded RNA-binding protein zn72d |  | DIV | 1.163 |  |
| AAEL008007 | conserved hypothetical protein |  | DIV | 1.162 |  |
| AAEL000430 | hypothetical protein |  | DIV | 1.158 |  |
| AAEL010665 | developmentally regulated RNA-binding protein |  | DIV | 1.148 |  |
| AAEL013057 | serine/threonine-protein kinase wnk 1,3,4 |  | DIV | 1.147 |  |
| AAEL002082 | latent nuclear antigen, putative |  | DIV | 1.143 |  |
| AAEL002090 | conserved hypothetical protein |  | DIV | 1.14 |  |
| AAEL004722 | GABA-A receptor interacting factor-1, putative |  | DIV | 1.136 |  |
| AAEL004041 | flotillin-2 |  | DIV | 1.132 |  |
| AAEL010676 | regulator of g protein signaling |  | DIV | 1.106 |  |
| AAEL008739 | shc transforming protein |  | DIV | 1.103 |  |
| AAEL011061 | hypothetical protein |  | DIV | 1.098 |  |
| AAEL007479 | hypothetical protein |  | DIV | 1.096 |  |
| AAEL005344 | ras association domain protein, putative |  | DIV | 1.093 |  |
| AAEL014851 | mediator complex subunit rgr-1 |  | DIV | 1.09 |  |
| AAEL005930 | ubiquitin-protein ligase |  | DIV | 1.088 |  |
| AAEL002277 | camp-dependent protein kinase type i-beta regulatory subunit |  | DIV | 1.082 |  |
| AAEL009422 | conserved hypothetical protein |  | DIV | 1.072 |  |
| AAEL006460 | par-6 gamma |  | DIV | 1.069 |  |
| AAEL001848 | conserved hypothetical protein |  | DIV | 1.066 |  |
| AAEL002607 | conserved hypothetical protein |  | DIV | 1.065 |  |
| AAEL000090 | secretory carrier-associated membrane protein (scamp) |  | DIV | 1.056 |  |
| AAEL005535 | conserved hypothetical protein |  | DIV | 1.054 |  |
| AAEL010344 | SEC14, putative |  | DIV | 1.051 |  |
| AAEL011006 | guanylate kinase |  | DIV | 1.042 |  |
| AAEL006539 | serine/threonine protein kinase |  | DIV | 1.04 |  |
| AAEL005284 | receptor tyrosine phosphatase type r2a |  | DIV | 1.033 |  |
| AAEL009495 | rab6-interacting |  | DIV | 1.032 |  |
| AAEL005400 | 2-hydroxyacid dehydrogenase |  | DIV | 1.027 |  |
| AAEL000395 | retinoid x receptor (rxr) |  | DIV | 1.025 |  |
| AAEL002175 | conserved hypothetical protein |  | DIV | 1.022 |  |
| AAEL010170 | raslated protein Rab-8A, putative |  | DIV | 1.018 |  |
| AAEL007889 | f-spondin |  | DIV | 1.015 |  |
| AAEL008078 | clk2 |  | DIV | 1.015 |  |
| AAEL014510 | sprouty |  | DIV | 1.009 |  |
| AAEL011417 | synaptojanin |  | DIV | 1.008 |  |
| AAEL000591 | hypothetical protein |  | DIV | 1.008 |  |
| AAEL001528 | hypothetical protein |  | DIV | 1.007 |  |
| AAEL005369 | zinc finger protein |  | DIV | 1.001 |  |
| AAEL010668 | quinone oxidoreductase |  | DIV | 0.998 |  |
| AAEL001099 | DEAD box polypeptide |  | DIV | 0.997 |  |
| AAEL002451 | zinc finger protein |  | DIV | 0.997 |  |
| AAEL003845 | Ets domain-containing protein |  | DIV | 0.993 |  |
| AAEL011970 | conserved hypothetical protein |  | DIV | 0.99 |  |
| AAEL007322 | phosphatidate phosphatase |  | DIV | 0.985 |  |
| AAEL010561 | conserved hypothetical protein |  | DIV | 0.984 |  |
| AAEL007841 | hypothetical protein |  | DIV | 0.982 |  |
| AAEL006780 | hypothetical protein |  | DIV | 0.98 |  |
| AAEL007436 | conserved hypothetical protein |  | DIV | 0.98 |  |
| AAEL000737 | rab6 gtpase activating protein, gapcena (rabgap1 protein) |  | DIV | 0.979 |  |
| AAEL001133 | conserved hypothetical protein |  | DIV | 0.975 |  |
| AAEL005683 | conserved hypothetical protein |  | DIV | 0.961 |  |
| AAEL007375 | pyruvate dehydrogenase |  | DIV | 0.957 |  |
| AAEL001393 | triple functional domain, trio |  | DIV | 0.95 |  |
| AAEL005238 | mck1 |  | DIV | 0.944 |  |
| AAEL009874 | conserved hypothetical protein |  | DIV | 0.944 |  |
| AAEL007362 | regulator of g protein signaling |  | DIV | 0.943 |  |
| AAEL001375 | Y-box binding protein |  | DIV | 0.942 |  |
| AAEL013308 | odd Oz protein |  | DIV | 0.94 |  |
| AAEL001398 | guanine nucleotide exchange factor |  | DIV | 0.94 |  |
| AAEL009171 | conserved hypothetical protein |  | DIV | 0.939 |  |
| AAEL004964 | hypothetical protein |  | DIV | 0.936 |  |
| AAEL009264 | hypothetical protein |  | DIV | 0.934 |  |
| AAEL001898 | conserved hypothetical protein |  | DIV | 0.933 |  |
| AAEL000421 | protein farnesyltransferase alpha subunit/rab geranylgeranyl transferase alpha subunit |  | DIV | 0.925 |  |
| AAEL012554 | maltose phosphorylase |  | DIV | 0.924 |  |
| AAEL000262 | conserved hypothetical protein |  | DIV | 0.922 |  |
| AAEL000770 | platelet-activating factor acetylhydrolase isoform 1b alpha subunit |  | DIV | 0.919 |  |
| AAEL003976 | conserved hypothetical protein |  | DIV | 0.915 |  |
| AAEL002937 | hypothetical protein |  | DIV | 0.915 |  |
| AAEL003540 | conserved hypothetical protein |  | DIV | 0.91 |  |
| AAEL000718 | virus-induced RNA, putative |  | DIV | 0.901 |  |
| AAEL005706 | triacylglycerol lipase |  | DIV | 0.901 |  |
| AAEL007662 | casein kinase |  | DIV | 0.9 |  |
| AAEL013619 | dolichyl-diphosphooligosaccharide protein glycosyltransferase |  | DIV | 0.898 |  |
| AAEL004209 | opioid-binding protein/cell adhesion molecule, putative |  | DIV | 0.897 |  |
| AAEL003750 | conserved hypothetical protein |  | DIV | 0.895 |  |
| AAEL004709 | protein phosphatase type 2c |  | DIV | 0.894 |  |
| AAEL009382 | conserved hypothetical protein |  | DIV | 0.884 |  |
| AAEL014999 | conserved hypothetical protein |  | DIV | 0.884 |  |
| AAEL012076 | conserved hypothetical protein |  | DIV | 0.882 |  |
| AAEL013334 | conserved hypothetical protein |  | DIV | 0.879 |  |
| AAEL005861 | vacuolar sorting protein (vps) |  | DIV | 0.878 |  |
| AAEL002251 | conserved hypothetical protein |  | DIV | 0.869 |  |
| AAEL009645 | hypothetical protein |  | DIV | 0.869 |  |
| AAEL000713 | reticulon/nogo |  | DIV | 0.862 |  |
| AAEL006651 | dystrophin |  | DIV | 0.86 |  |
| AAEL012999 | mixed lineage kinase |  | DIV | 0.86 |  |
| AAEL009606 | conserved hypothetical protein |  | DIV | 0.857 |  |
| AAEL008591 | zinc finger protein, putative |  | DIV | 0.856 |  |
| AAEL013459 | conserved hypothetical protein |  | DIV | 0.851 |  |
| AAEL006041 | conserved hypothetical protein |  | DIV | 0.848 |  |
| AAEL013510 | smaug protein |  | DIV | 0.848 |  |
| AAEL005528 | conserved hypothetical protein |  | DIV | 0.847 |  |
| AAEL003824 | conserved hypothetical protein |  | DIV | 0.843 |  |
| AAEL011575 | conserved hypothetical protein |  | DIV | 0.839 |  |
| AAEL006990 | conserved hypothetical protein |  | DIV | 0.836 |  |
| AAEL002306 | hect E3 ubiquitin ligase |  | DIV | 0.835 |  |
| AAEL013068 | protein phsophatase-2a |  | DIV | 0.833 |  |
| AAEL005320 | skeletrophin |  | DIV | 0.832 |  |
| AAEL000079 | hypothetical protein |  | DIV | 0.831 |  |
| AAEL010020 | mediator complex subunit rgr-1 |  | DIV | 0.829 |  |
| AAEL007011 | conserved hypothetical protein |  | DIV | 0.828 |  |
| AAEL000399 | conserved hypothetical protein |  | DIV | 0.827 |  |
| AAEL007682 | conserved hypothetical protein |  | DIV | 0.826 |  |
| AAEL001919 | protein tyrosine phosphatase, nonceptor type nt1 |  | DIV | 0.822 |  |
| AAEL005302 | beta-1,4-galactosyltransferase |  | DIV | 0.822 |  |
| AAEL007065 | adpbosylation factor, arf |  | DIV | 0.82 |  |
| AAEL003509 | smap1 |  | DIV | 0.816 |  |
| AAEL003955 | hypothetical protein |  | DIV | 0.816 |  |
| AAEL003928 | pdgf/vegf receptor |  | DIV | 0.813 |  |
| AAEL000824 | hypothetical protein |  | DIV | 0.812 |  |
| AAEL004472 | hypothetical protein |  | DIV | 0.812 |  |
| AAEL010750 | hypothetical protein |  | DIV | 0.809 |  |
| AAEL005069 | raslated protein Rab-1A, putative |  | DIV | 0.808 |  |
| AAEL006806 | atrial natriuretic peptide receptor |  | DIV | 0.807 |  |
| AAEL002706 | hypothetical protein |  | DIV | 0.807 |  |
| AAEL007884 | conserved membrane protein at 44E, putative |  | DIV | 0.803 |  |
| AAEL008107 | f14p3.9 protein (auxin transport protein) |  | DIV | 0.802 |  |
| AAEL000857 | conserved hypothetical protein |  | DIV | 0.801 |  |
| AAEL014931 | sarm1 |  | DIV | 0.796 |  |
| AAEL001709 | adaptin, alpha/gamma/epsilon |  | DIV | 0.796 |  |
| AAEL008733 | histidine triad (hit) protein member |  | DIV | 0.791 |  |
| AAEL005502 | conserved hypothetical protein |  | DIV | 0.79 |  |
| AAEL001640 | multicopper oxidase |  | DIV | 0.789 |  |
| AAEL003799 | conserved hypothetical protein |  | DIV | 0.787 |  |
| AAEL002142 | conserved hypothetical protein |  | DIV | 0.786 |  |
| AAEL015466 | conserved hypothetical protein |  | DIV | 0.785 |  |
| AAEL007687 | transmembrane 9 superfamily protein member 4 |  | DIV | 0.785 |  |
| AAEL013280 | rho guanine exchange factor |  | DIV | 0.785 |  |
| AAEL003454 | Phocein protein, putative |  | DIV | 0.783 |  |
| AAEL001152 | beta-1,3-galactosyltransferase-6 |  | DIV | 0.782 |  |
| AAEL008793 | conserved hypothetical protein |  | DIV | 0.782 |  |
| AAEL007455 | thrombospondin |  | DIV |  | 3.298 |
| AAEL007212 | expressed protein (HR96) |  | DIV |  | 1.782 |
| AAEL002611 | conserved hypothetical protein |  | DIV |  | 1.747 |
| AAEL010218 | GATA transcription factor (GATAa) |  | DIV |  | 1.433 |
| AAEL013072 | conserved hypothetical protein |  | DIV |  | 1.419 |
| AAEL007370 | conserved hypothetical protein |  | DIV |  | 1.402 |
| AAEL002732 | nephrin |  | DIV |  | 1.333 |
| AAEL002364 | hypothetical protein |  | DIV |  | 1.329 |
| AAEL007665 | hypothetical protein |  | DIV |  | 1.254 |
| AAEL011572 | Oct1, putative |  | DIV |  | 1.231 |
| AAEL002637 | tripartite motif protein trim9 |  | DIV |  | 1.2 |
| AAEL011623 | conserved hypothetical protein |  | DIV |  | 1.18 |
| AAEL014622 | conserved hypothetical protein |  | DIV |  | 1.172 |
| AAEL015487 | zinc finger protein, putative |  | DIV |  | 1.135 |
| AAEL010229 | hypothetical protein |  | DIV |  | 1.068 |
| AAEL011327 | nuclear transcription factor 4, putative |  | DIV |  | 1.067 |
| AAEL004412 | polo kinase kinase |  | DIV |  | 1.052 |
| AAEL003902 | d(1a,b) dopamine receptor |  | DIV |  | 1.049 |
| AAEL002448 | hypothetical protein |  | DIV |  | 1.045 |
| AAEL001388 | hypothetical protein |  | DIV |  | 1.031 |
| AAEL012998 | conserved hypothetical protein |  | DIV |  | 1.029 |
| AAEL013231 | hypothetical protein |  | DIV |  | 1.019 |
| AAEL010062 | conserved hypothetical protein |  | DIV |  | 0.933 |
| AAEL007199 | hypothetical protein |  | DIV |  | 0.9 |
| AAEL005109 | wdpeat protein |  | DIV |  | 0.893 |
| AAEL003312 | hypothetical protein |  | DIV |  | 0.869 |
| AAEL013430 | hypothetical protein |  | DIV |  | 0.865 |
| AAEL003508 | serine-pyruvate aminotransferase |  | DIV |  | 0.864 |
| AAEL002120 | zinc finger protein |  | DIV |  | 0.837 |
| AAEL004508 | hypothetical protein |  | DIV |  | 0.836 |
| AAEL012570 | hypothetical protein |  | DIV |  | 0.829 |
| AAEL009903 | hypothetical protein |  | DIV |  | 0.825 |
| AAEL001569 | conserved hypothetical protein |  | DIV |  | 0.805 |
| AAEL001094 | conserved hypothetical protein |  | DIV |  | 0.792 |
| AAEL000165 | conserved hypothetical protein |  | DIV |  | 0.782 |
| AAEL012086 | hypothetical protein |  | DIV | -1.706 | -1.378 |
| AAEL009520 | hypothetical protein |  | DIV | -1.978 | -1.272 |
| AAEL000703 | glycogen phosphorylase |  | DIV | -0.821 | -1.233 |
| AAEL007677 | phospholysine phosphohistidine inorganic pyrophosphate phosphatase |  | DIV | -0.971 | -1.121 |
| AAEL011220 | Ati or CPXV158 protein, putative |  | DIV | -1.407 | -1.087 |
| AAEL001635 | conserved hypothetical protein |  | DIV | -0.995 | -0.989 |
| AAEL004139 | hypothetical protein |  | DIV | -0.844 | -0.989 |
| AAEL000541 | fasciclin, putative |  | DIV | -1.096 | -0.944 |
| AAEL005216 | conserved hypothetical protein |  | DIV | -1.133 | -0.909 |
| AAEL004221 | glycogen synthase |  | DIV | -0.87 | -0.893 |
| AAEL004150 | fibrinogen and fibronectin |  | DIV | -0.907 | -0.889 |
| AAEL012187 | lethal(3)malignant brain tumor |  | DIV | -1.001 | -0.863 |
| AAEL003651 | conserved hypothetical protein |  | DIV | -0.91 | -0.824 |
| AAEL003729 | conserved hypothetical protein |  | DIV | -1.064 | -0.813 |
| AAEL013453 | sarcolemmal associated protein, putative |  | DIV | -0.792 | -0.813 |
| AAEL001650 | conserved hypothetical protein |  | DIV | -1.046 | -0.808 |
| AAEL002569 | serine/threonine kinase |  | DIV | -0.893 | -0.803 |
| AAEL012238 | glutaredoxin, putative |  | DIV | -0.788 | -0.784 |
| AAEL004229 | glutathione transferase AtGST, putative |  | DIV | -0.87 | -0.781 |
| AAEL011596 | mitotic checkpoint serine/threonine-protein kinase bub1 and bubr1 |  | DIV | -1.062 | -0.778 |
| AAEL006207 | conserved hypothetical protein |  | DIV | -1.182 |  |
| AAEL014596 | hypothetical protein |  | DIV | -1.161 |  |
| AAEL012391 | conserved hypothetical protein |  | DIV | -1.146 |  |
| AAEL013974 | conserved hypothetical protein |  | DIV | -1.106 |  |
| AAEL008719 | Sm protein G, putative |  | DIV | -1.104 |  |
| AAEL008316 | mitotic spindle assembly checkpoint protein mad2 |  | DIV | -1.062 |  |
| AAEL008646 | fibrinogen and fibronectin |  | DIV | -1.036 |  |
| AAEL011235 | conserved hypothetical protein |  | DIV | -0.994 |  |
| AAEL008716 | conserved hypothetical protein |  | DIV | -0.987 |  |
| AAEL008605 | inosine triphosphate pyrophosphatase (itpase) (inosine triphosphatase) |  | DIV | -0.987 |  |
| AAEL015555 | conserved hypothetical protein |  | DIV | -0.978 |  |
| AAEL012628 | conserved hypothetical protein |  | DIV | -0.975 |  |
| AAEL000465 | conserved hypothetical protein |  | DIV | -0.947 |  |
| AAEL008369 | acylphosphatase, putative |  | DIV | -0.946 |  |
| AAEL004512 | zinc finger protein |  | DIV | -0.92 |  |
| AAEL005557 | hypothetical protein |  | DIV | -0.916 |  |
| AAEL001653 | fetal globin-inducing factor |  | DIV | -0.912 |  |
| AAEL002744 | hypothetical protein |  | DIV | -0.912 |  |
| AAEL010622 | hypothetical protein |  | DIV | -0.907 |  |
| AAEL007907 | serine/threonine protein kinase |  | DIV | -0.891 |  |
| AAEL010013 | wdpeat protein |  | DIV | -0.881 |  |
| AAEL002739 | conserved hypothetical protein |  | DIV | -0.879 |  |
| AAEL011834 | hypothetical protein |  | DIV | -0.875 |  |
| AAEL000147 | single-stranded DNA binding protein, putative |  | DIV | -0.854 |  |
| AAEL013943 | mediator complex, 100kD-subunit, putative |  | DIV | -0.848 |  |
| AAEL003888 | ubiquitin |  | DIV | -0.846 |  |
| AAEL005976 | adenine phosphoribosyltransferase, putative |  | DIV | -0.845 |  |
| AAEL001838 | conserved hypothetical protein |  | DIV | -0.843 |  |
| AAEL000425 | conserved hypothetical protein |  | DIV | -0.838 |  |
| AAEL015060 | Rad51A protein, putative |  | DIV | -0.835 |  |
| AAEL015658 | conserved hypothetical protein |  | DIV | -0.834 |  |
| AAEL004086 | aldo-keto reductase |  | DIV | -0.83 |  |
| AAEL009701 | conserved hypothetical protein |  | DIV | -0.829 |  |
| AAEL011362 | hypothetical protein |  | DIV | -0.825 |  |
| AAEL007395 | conserved hypothetical protein |  | DIV | -0.822 |  |
| AAEL007564 | zinc finger protein |  | DIV | -0.817 |  |
| AAEL002888 | williams-beuren syndrome critical region protein |  | DIV | -0.812 |  |
| AAEL012771 | conserved hypothetical protein |  | DIV | -0.811 |  |
| AAEL009149 | kinectin, putative |  | DIV | -0.811 |  |
| AAEL009425 | hypothetical protein |  | DIV | -0.809 |  |
| AAEL012938 | zinc finger protein |  | DIV | -0.803 |  |
| AAEL008075 | Multiple PDZ domain protein |  | DIV | -0.793 |  |
| AAEL005719 | cleavage stimulation factor |  | DIV | -0.785 |  |
| AAEL011604 | perlecan |  | DIV | -0.785 |  |
| AAEL013844 | diazepam binding inhibitor, putative |  | DIV | -0.782 |  |
| AAEL006787 | conserved hypothetical protein |  | DIV | -0.782 |  |
| AAEL004779 | cyclophilin-10 |  | DIV | -0.779 |  |
| AAEL006948 | tomosyn |  | DIV | -0.779 |  |
| AAEL004335 | secreted ferritin G subunit precursor, putative |  | DIV | -0.776 |  |
| AAEL014192 | conserved hypothetical protein |  | DIV |  | -1.034 |
| AAEL014438 | Juvenile hormone-inducible protein, putative |  | DIV |  | -1.197 |
| AAEL011606 | conserved hypothetical protein |  | DIV |  | -1.165 |
| AAEL008486 | protein kinase C inhibitor, putative |  | DIV |  | -1.049 |
| AAEL006628 | conserved hypothetical protein |  | DIV |  | -1.013 |
| AAEL000065 | conserved hypothetical protein |  | DIV |  | -0.988 |
| AAEL005297 | guanine nucleotide exchange factor |  | DIV |  | -0.902 |
| AAEL013338 | lethal(2)essential for life protein, l2efl |  | DIV |  | -0.901 |
| AAEL015636 | interleukin enhancer binding factor |  | DIV |  | -0.892 |
| AAEL010472 | helix-loop-helix protein hen |  | DIV |  | -0.885 |
| AAEL002950 | conserved hypothetical protein |  | DIV |  | -0.879 |
| AAEL005395 | conserved hypothetical protein |  | DIV |  | -0.871 |
| AAEL000629 | adenylate kinase 3, putative |  | DIV |  | -0.853 |
| AAEL004004 | chromatin regulatory protein sir2 |  | DIV |  | -0.842 |
| AAEL011816 | conserved hypothetical protein |  | DIV |  | -0.834 |
| AAEL002399 | aspartate aminotransferase |  | DIV |  | -0.827 |
| AAEL006203 | Juvenile hormone-inducible protein, putative |  | DIV |  | -0.819 |
| AAEL015017 | islet cell autoantigen |  | DIV |  | -0.812 |
| AAEL013644 | ubiquitously transcribed sex (x/y) chromosome tetratricopeptide repeat protein |  | DIV |  | -0.806 |
| AAEL006965 | NBP2b protein, putative |  | DIV |  | -0.806 |
| AAEL004566 | myo inositol monophosphatase |  | DIV |  | -0.799 |
| AAEL012939 | gamma-subunit,methylmalonyl-CoA decarboxylase, putative |  | DIV |  | -0.778 |
| AAEL001703 | serine-type enodpeptidase, putative |  | DIG | -1.281 | -1.12 |
| AAEL002273 | Trypsin, putative |  | DIG | -0.986 |  |
| AAEL010951 | glutamate decarboxylase |  | IMM | 0.814 | 0.894 |
| AAEL007363 | leucinech transmembrane protein |  | IMM | 1.055 | 0.837 |
| Aaeg:N49982 | CLIPB16 | CLIPB16 | IMM | 0.778 | 1.526 |
| AAEL007613 | toll | TOLL1A | IMM | 1.179 |  |
| AAEL002166 | leucine rich repeat (in flii) interacting protein |  | IMM | 1.131 |  |
| Aaeg:N41501 | CAT1A | CAT1A | IMM | 1.101 |  |
| AAEL002206 | rap gtpase-activating protein | GALE | IMM | 1.043 |  |
| AAEL005832 | programmed cell death |  | IMM | 0.969 |  |
| AAEL000709 | developmental protein cactus | CACT | IMM | 0.929 |  |
| AAEL003119 | conserved hypothetical protein | CTL6 | IMM | 0.879 |  |
| Aaeg:N42080 | DDC | DDC | IMM | 0.867 |  |
| AAEL014989 | peptidoglycan recognition protein-1, putative | PGRPLD | IMM | 0.855 |  |
| AAEL014356 | conserved hypothetical protein | CTLSE2 | IMM | 0.843 |  |
| AAEL003554 | leucine rich repeat protein |  | IMM | 0.805 |  |
| Aaeg:N51900 | HSC70-3 | HSC70-3 | IMM | 0.789 |  |
| AAEL001914 | scavenger receptor, putative | SCRAC1 | IMM | 0.78 |  |
| AAEL006702 | fibrinogen and fibronectin | FREP33 | IMM |  | 1.91 |
| Aaeg:N31575 | CTLMA15 | CTLMA15 | IMM |  | 1.749 |
| AAEL011455 | galactose-specific C-type lectin, putative | CTLMA12 | IMM |  | 1.622 |
| AAEL006699 | fibrinogen and fibronectin | FREP34 | IMM |  | 1.546 |
| AAEL011764 | prophenoloxidase | PPO10 | IMM |  | 1.537 |
| AAEL006137 | conserved hypothetical protein | SRPN19 | IMM |  | 1.475 |
| Aaeg:N52152 | SOCS | SOCS | IMM |  | 1.448 |
| Aaeg:N18089 | DCE | DCE | IMM |  | 1.409 |
| AAEL009420 | cd36 antigen | SCRBQ1 | IMM |  | 1.389 |
| AAEL013417 | fibrinogen and fibronectin | FREP24 | IMM |  | 1.376 |
| Aaeg:N12802 | WASP | WASP | IMM |  | 1.334 |
| Aaeg:N32065 | CTL | CTL | IMM |  | 1.315 |
| AAEL000533 | antifreeze protein, putative | CTL16 | IMM |  | 1.314 |
| Aaeg:N6593 | DSCAM | DSCAM | IMM |  | 1.311 |
| AAEL002354 | oxidase/peroxidase | HPX5 | IMM |  | 1.271 |
| Aaeg:N23036 | CLIP | CLIP | IMM |  | 1.242 |
| AAEL011619 | galactose-specific C-type lectin, putative | CTLGA8 | IMM |  | 1.238 |
| AAEL002704 | serine protease inhibitor (serpin-4), putative | SRPN23 | IMM |  | 1.149 |
| AAEL000633 | toll | TOLL8 | IMM |  | 1.144 |
| Aaeg:N35123 | HPX | HPX | IMM |  | 1.101 |
| AAEL010992 | conserved hypothetical protein | CTL8 | IMM |  | 1.083 |
| AAEL008681 | conserved hypothetical protein | CTL12 | IMM |  | 1.048 |
| Aaeg:N38609 | RANK1 | RANK1 | IMM |  | 1.044 |
| AAEL009551 | toll | TOLL11 | IMM |  | 1.042 |
| Aaeg:N40789 | SOCS | SOCS | IMM |  | 0.897 |
| Aaeg:N44241 | GPX | GPX | IMM |  | 0.857 |
| AAEL009176 | gram-negative bacteria binding protein | GNBPB3 | IMM |  | 0.832 |
| AAEL007768 | myd88 | MYD88 | IMM |  | 0.818 |
| AAEL007696 | embryonic polarity dorsal (REL1A) | REL1A | IMM |  | 0.807 |
| AAEL000227 | epithelial membrane protein | SCRB8 | IMM |  | 0.803 |
| Aaeg:N44512 | DCE | DCE | IMM | -2.526 | -1.864 |
| AAEL000598 | antibacterial peptide, putative | CECD | IMM | -1.176 | -1.144 |
| AAEL000611 | antibacterial peptide, putative | CECE | IMM | -1.547 | -1.065 |
| AAEL003832 | conserved hypothetical protein | DEFC | IMM | -0.992 | -1.016 |
| AAEL001163 | macroglobulin/complement | TEP23 | IMM | -1.076 | -1.001 |
| AAEL015515 | antibacterial peptide, putative | CECG | IMM | -1.323 | -0.942 |
| AAEL009474 | peptidoglycan recognition protein-lc isoform | PGRPS1 | IMM | -0.992 | -0.93 |
| AAEL011009 | fibrinogen and fibronectin | FREP13 | IMM | -1.096 | -0.899 |
| AAEL009384 | fibrinogen and fibronectin | FREP5 | IMM | -1.144 | -0.787 |
| AAEL000625 | antibacterial peptide, putative | CECF | IMM | -1.048 |  |
| AAEL005800 | serine protease, putative | CLIPE11 | IMM | -1.037 |  |
| AAEL007107 | serine protease, putative |  | IMM | -1.027 |  |
| AAEL002601 | serine protease, putative | CLIPA1 | IMM | -0.907 |  |
| AAEL007626 | gram-negative bacteria binding protein | GNBPA1 | IMM | -0.906 |  |
| Aaeg:N31904 | CTLMA14 | CTLMA14 | IMM | -0.877 |  |
| AAEL003632 | clip-domain serine protease, putative | CLIPB39 | IMM | -0.874 |  |
| AAEL011607 | galactose-specific C-type lectin, putative | CTLMA14 | IMM | -0.848 |  |
| AAEL014251 | conserved hypothetical protein | IAP5 | IMM | -0.837 |  |
| AAEL000621 | antibacterial peptide, putative | CECN | IMM | -0.828 |  |
| AAEL006161 | clip-domain serine protease, putative | CLIPB31 | IMM | -0.81 |  |
| AAEL003857 | conserved hypothetical protein | DEFD | IMM | -0.777 |  |
| AAEL000627 | antibacterial peptide, putative | CECA | IMM |  | -1.19 |
| AAEL004868 | hemomucin |  | IMM |  | -0.811 |
| AAEL009842 | keratinocyte lectin, putative | GALE12 | IMM |  | -0.957 |
| AAEL014246 | glucosyl/glucuronosyl transferases |  | MET | 1.062 | 1.31 |
| AAEL002688 | glucosyl/glucuronosyl transferases |  | MET | 0.905 | 0.862 |
| AAEL013128 | elongase, putative |  | MET | 1.561 |  |
| AAEL014664 | AMP dependent coa ligase |  | MET | 1.355 |  |
| AAEL001273 | Sec24B protein, putative |  | MET | 1.271 |  |
| AAEL013458 | glutamine synthetase 1, 2 (glutamate-amonia ligase) (gs) |  | MET | 1.268 |  |
| AAEL010256 | E3 ubiquitin ligase |  | MET | 1.173 |  |
| AAEL006687 | exportin |  | MET | 1.17 |  |
| AAEL014871 | methylenetetrahydrofolate dehydrogenase |  | MET | 1.063 |  |
| AAEL002430 | n-acetylglucosamine-6-phosphate deacetylase |  | MET | 1.061 |  |
| AAEL010751 | methylenetetrahydrofolate dehydrogenase |  | MET | 1.037 |  |
| AAEL004952 | protein N-terminal asparagine amidohydrolase, putative |  | MET | 1.003 |  |
| AAEL008374 | E3 ubiquitin-protein ligase nedd-4 |  | MET | 0.996 |  |
| AAEL008687 | tar RNA binding protein (trbp) |  | MET | 0.973 |  |
| AAEL004294 | dihydrolipoamide acetyltransferase component of pyruvate dehydrogenase |  | MET | 0.963 |  |
| AAEL005763 | lysosomal alpha-mannosidase (mannosidase alpha class 2b member 1) |  | MET | 0.962 |  |
| AAEL008507 | srpk |  | MET | 0.932 |  |
| AAEL012409 | pantothenate kinase |  | MET | 0.917 |  |
| AAEL001593 | glycerol-3-phosphate dehydrogenase |  | MET | 0.887 |  |
| AAEL004865 | cyclin g |  | MET | 0.882 |  |
| AAEL003402 | sphingomyelin phosphodiesterase |  | MET | 0.871 |  |
| AAEL003091 | glucosyl/glucuronosyl transferases |  | MET | 0.868 |  |
| AAEL008393 | phosphatidylserine synthase |  | MET | 0.826 |  |
| AAEL001523 | secretory Phospholipase A2, putative |  | MET | 0.825 |  |
| AAEL014965 | nova |  | MET | 0.817 |  |
| AAEL005380 | mixed-lineage leukemia protein, mll |  | MET | 0.815 |  |
| AAEL003873 | glycerol-3-phosphate dehydrogenase |  | MET | 0.801 |  |
| AAEL004757 | cleavage and polyadenylation specificity factor |  | MET | 0.8 |  |
| AAEL002528 | histone deacetylase |  | MET | 0.8 |  |
| AAEL000690 | steroid dehydrogenase |  | MET |  | 3.151 |
| AAEL011957 | elongase, putative |  | MET |  | 1.025 |
| AAEL012446 | survivin |  | MET |  | 0.971 |
| AAEL000006 | phosphoenolpyruvate carboxykinase |  | MET |  | 0.859 |
| AAEL013525 | Timp-3, putative |  | MET |  | 0.848 |
| AAEL002658 | AMP dependent ligase |  | MET |  | 0.822 |
| AAEL013831 | pyrroline-5-carboxylate dehydrogenase |  | MET |  | 0.809 |
| AAEL002542 | triosephosphate isomerase |  | MET | -1.113 | -1.009 |
| AAEL012014 | l-lactate dehydrogenase |  | MET | -1.069 | -0.966 |
| AAEL012418 | deoxyribonuclease ii |  | MET | -1.046 | -0.963 |
| AAEL009237 | glycoside hydrolases |  | MET | -0.856 | -0.801 |
| AAEL011233 | SM protein G, putative |  | MET | -1.359 |  |
| AAEL012994 | glucose-6-phosphate isomerase |  | MET | -1.036 |  |
| AAEL012455 | alcohol dehydrogenase |  | MET | -0.895 |  |
| AAEL015020 | glycoside hydrolases |  | MET | -0.87 |  |
| AAEL004778 | acyl-coa dehydrogenase |  | MET | -0.857 |  |
| AAEL008865 | oligoribonuclease, mitochondrial |  | MET | -0.833 |  |
| AAEL014287 | deoxycytidylate deaminase |  | MET | -0.818 |  |
| AAEL007893 | short chain type dehydrogenase |  | MET | -0.798 |  |
| AAEL014139 | proacrosin, putative |  | MET |  | -0.94 |
| AAEL008668 | MASP-2 protein, putative |  | MET |  | -0.839 |
| AAEL008124 | POSSIBLE RNA METHYLTRANSFERASE, putative |  | MET |  | -0.787 |
| AAEL014353 | conserved hypothetical protein |  | PROT | 0.98 | 1.088 |
| AAEL003026 | regulator of g protein signaling |  | PROT | 1.598 |  |
| AAEL002663 | kuzbanian |  | PROT | 1.344 |  |
| AAEL008202 | serine-type enodpeptidase, putative |  | PROT | 1.209 |  |
| AAEL004138 | signal peptide peptidase |  | PROT | 1.06 |  |
| AAEL004980 | conserved hypothetical protein |  | PROT | 0.952 |  |
| AAEL012335 | conserved hypothetical protein |  | PROT | 0.931 |  |
| AAEL003733 | hypothetical protein |  | PROT | 0.821 |  |
| AAEL001540 | ubiquitin specific protease |  | PROT | 0.808 |  |
| AAEL003965 | calpain, putative |  | PROT | 0.789 |  |
| AAEL014350 | hypothetical protein |  | PROT |  | 1.501 |
| AAEL015257 | hypothetical protein |  | PROT |  | 1.063 |
| AAEL006542 | retinoid-inducible serine carboxypeptidase (serine carboxypeptidase |  | PROT |  | 0.775 |
| AAEL013605 | hypothetical protein |  | PROT | -1.358 | -1.359 |
| AAEL005107 | hypothetical protein |  | PROT | -1.403 | -1.18 |
| AAEL015272 | zinc carboxypeptidase |  | PROT | -1.219 | -1.18 |
| AAEL008769 | serine-type enodpeptidase, putative |  | PROT | -1.365 |  |
| AAEL003967 | calpain, putative |  | PROT | -0.794 |  |
| AAEL010989 | hypothetical protein |  | RSM | 1.511 | 0.79 |
| AAEL005342 | conserved hypothetical protein |  | RSM | 0.861 |  |
| AAEL009076 | conserved hypothetical protein |  | RSM |  | 0.853 |
| AAEL011850 | cytochrome P450 |  | RSM | 1.63 |  |
| AAEL006386 | mitochondrial 39S ribosomal protein L39 |  | RSM | 1.5 |  |
| AAEL010226 | daughterless |  | RSM | 1.151 |  |
| AAEL004589 | small calcium-binding mitochondrial carrier, putative |  | RSM | 1.118 |  |
| AAEL014608 | cytochrome P450 |  | RSM | 0.935 |  |
| AAEL007235 | mitochondrial uncoupling protein |  | RSM | 0.934 |  |
| AAEL003215 | heat shock factor binding protein, putative |  | RSM | 0.932 |  |
| AAEL010546 | heat shock factor binding protein, putative |  | RSM | 0.873 |  |
| AAEL000895 | peroxisome biogenesis factor 1 (peroxin-1) |  | RSM | 0.824 |  |
| AAEL001024 | mitochondrial carrier protein |  | RSM | 0.788 |  |
| AAEL006318 | short-chain dehydrogenase |  | RSM |  | 1.647 |
| AAEL013350 | heat shock protein 26kD, putative |  | RSM | -1.91 | -1.532 |
| AAEL007046 | mitochondrial brown fat uncoupling protein |  | RSM | -1.08 | -1.452 |
| AAEL010372 | aldehyde oxidase |  | RSM | -1.204 | -0.993 |
| AAEL013693 | excision repair cross-complementing 1 ercc1 |  | RSM | -0.898 | -0.954 |
| AAEL012308 | aldehyde oxidase |  | RSM | -1.172 | -0.912 |
| AAEL003195 | carboxylesterase |  | RSM | -0.937 | -0.906 |
| AAEL010677 | oxidoreductase |  | RSM | -1.212 | -0.898 |
| AAEL010380 | aldehyde oxidase |  | RSM | -0.908 | -0.88 |
| AAEL002523 | mitochondrial inner membrane protein translocase, 9kD-subunit, putative |  | RSM | -1.122 |  |
| AAEL002486 | mitochondrial inner membrane protein translocase, 9kD-subunit, putative |  | RSM | -1.026 |  |
| AAEL004829 | NADH dehydrogenase, putative |  | RSM | -0.916 |  |
| AAEL011752 | glutathione-s-transferase theta, gst |  | RSM | -0.907 |  |
| AAEL006984 | cytochrome P450 |  | RSM | -0.899 |  |
| AAEL005508 | NADH-ubiquinone oxidoreductase 24 kda subunit |  | RSM | -0.898 |  |
| AAEL007355 | mitochondrial ribosomal protein, S18A, putative |  | RSM | -0.89 |  |
| AAEL003770 | conserved hypothetical protein |  | RSM | -0.817 |  |
| AAEL002783 | mitochondrial ribosomal protein, L37, putative |  | RSM | -0.796 |  |
| AAEL004450 | cytochrome b5, putative |  | RSM | -0.796 |  |
| AAEL008601 | mitochondrial ribosomal protein, L28, putative |  | RSM | -0.794 |  |
| AAEL007946 | glutathione-s-transferase theta, gst |  |  |  | -0.907 |
| AAEL013790 | mitochondrial ribosomal protein, L50, putative |  |  |  | -0.86 |
| AAEL005113 | alpha-esterase |  |  |  | -0.84 |
| AAEL012584 | DNA topoisomerase/gyrase |  | RTT | 1.165 |  |
| AAEL004716 | chromodomain helicase DNA binding protein |  | RTT | 1.111 |  |
| AAEL007923 | eukaryotic translation initiation factor 4 gamma |  | RTT | 1.09 |  |
| AAEL013135 | chromodomain helicase DNA binding protein |  | RTT | 1.069 |  |
| AAEL010467 | heterogeneous nuclear ribonucleoprotein |  | RTT | 1.044 |  |
| AAEL004119 | ribonuclease p/mrp subunit |  | RTT | 1.027 |  |
| AAEL013653 | tata-box binding protein |  | RTT | 0.987 |  |
| AAEL010222 | transcription factor GATA-4 (GATA binding factor-4) |  | RTT | 0.883 |  |
| AAEL015263 | RNA and export factor binding protein |  | RTT | 0.876 |  |
| AAEL002853 | ccaat/enhancer binding protein |  | RTT | 0.862 |  |
| AAEL003800 | 5'-3' exoribonuclease, putative |  | RTT | 0.86 |  |
| AAEL002551 | DNA topoisomerase type I |  | RTT | 0.859 |  |
| AAEL008738 | DEAD box ATP-dependent RNA helicase |  | RTT | 0.807 |  |
| AAEL000193 | histone-lysine n-methyltransferase |  | RTT | 0.777 |  |
| AAEL005127 | ribonuclease UK114, putative |  | RTT |  | 1.464 |
| AAEL001912 | forkhead protein/ forkhead protein domain |  | RTT |  | 0.89 |
| AAEL002359 | homeobox protein onecut |  | RTT |  | 0.889 |
| AAEL006473 | arginine/serinech splicing factor |  | RTT | -0.908 | -1.097 |
| AAEL007801 | exonuclease |  | RTT | -1.063 | -0.907 |
| AAEL004848 | small nuclear ribonucleoprotein, core, putative |  | RTT | -1.274 | -0.894 |
| AAEL003985 | small nuclear ribonucleoprotein, core, putative |  | RTT | -0.783 | -0.879 |
| AAEL010642 | poly(A)-binding protein, putative |  | RTT | -0.971 | -0.818 |
| AAEL014169 | small nuclear ribonucleoprotein, core, putative |  | RTT | -1.255 | -0.814 |
| AAEL001280 | 28S ribosomal protein S15, mitochondrial precursor |  | RTT | -0.902 |  |
| AAEL015236 | signal recognition particle, 9kD-subunit, putative |  | RTT | -0.861 |  |
| AAEL015045 | transcription factor IIIA, putative |  | RTT | -0.818 |  |
| AAEL001363 | small nuclear ribonucleoprotein Sm D1, putative |  | RTT | -0.8 |  |
| AAEL005888 | DNA polymerase theta |  | RTT | -0.791 |  |
| AAEL007885 | translation initiation factor-3 (IF3), putative |  | RTT |  | -1.029 |
| AAEL006582 | calcium-transporting atpase sarcoplasmic/endoplasmic reticulum type (calcium pump) |  | TRP | 1.829 |  |
| AAEL005392 | dihydropyridine-sensitive l-type calcium channel |  | TRP | 1.453 |  |
| AAEL003393 | ATP synthase beta subunit |  | TRP | 1.321 |  |
| AAEL008928 | inwardly rectifying k+ channel, putative |  | TRP | 1.287 |  |
| AAEL012657 | syntaxin, putative |  | TRP | 1.105 |  |
| AAEL010361 | rer1 protein |  | TRP | 1.066 |  |
| AAEL005043 | ATP-dependent bile acid permease |  | TRP | 1.004 |  |
| AAEL010470 | calcineurin b subunit |  | TRP | 0.995 |  |
| AAEL004141 | phosphatidylinositol transfer protein/retinal degeneration b protein |  | TRP | 0.988 |  |
| AAEL011657 | importin alpha |  | TRP | 0.962 |  |
| AAEL007971 | tyrosine transporter |  | TRP | 0.935 |  |
| AAEL009088 | liquid facets |  | TRP | 0.911 |  |
| AAEL000567 | sugar transporter |  | TRP | 0.908 |  |
| AAEL003789 | Exportin, putative |  | TRP | 0.868 |  |
| AAEL010608 | succinate dehydrogenase |  | TRP | 0.841 |  |
| AAEL013704 | beta-arrestin 1, putative |  | TRP | 0.813 |  |
| AAEL013614 | clathrin heavy chain |  | TRP | 0.81 |  |
| AAEL002061 | cation-transporting atpase 13a1 (g-box binding protein) |  | TRP | 0.808 |  |
| AAEL000417 | monocarboxylate transporter |  | TRP | 0.797 |  |
| AAEL004743 | multidrug resistance protein 2 (atp-binding cassette protein c) |  | TRP | 0.793 |  |
| AAEL002412 | monocarboxylate transporter |  | TRP | 0.789 |  |
| AAEL008587 | glutamate receptor, ionotropic, n-methyl d-aspartate |  | TRP |  | 1.25 |
| AAEL010481 | sugar transporter |  | TRP |  | 1.121 |
| AAEL006047 | histamine-gated chloride channel subunit |  | TRP |  | 0.92 |
| AAEL010823 | ATP synthase delta chain |  | TRP | -0.969 | -1.117 |
| AAEL004025 | glucose dehydrogenase |  | TRP | -1.051 | -1.025 |
| AAEL003626 | sodium/shloride dependent amino acid transporter |  | TRP | -1.301 | -0.896 |
| AAEL005859 | amino acid transporter |  | TRP | -1.019 |  |
| AAEL000435 | THO complex, putative |  | TRP | -0.903 |  |
| AAEL004620 | sorting nexin |  | TRP |  | -0.893 |
| AAEL011423 | sugar transporter |  | TRP |  | -1.093 |
| AAEL013215 | sulfonylurea receptor/ abc transporter |  | TRP |  | -0.966 |
| AAEL005249 | abc transporter |  | TRP |  | -0.79 |
| AAEL001313 | conserved hypothetical protein |  | UNK | 0.965 | 0.805 |
| AAEL003223 | hypothetical protein |  | UNK | 1.832 | 0.972 |
| AAEL003025 | hypothetical protein |  | UNK | 1.381 | 1.018 |
| AAEL002470 | hypothetical protein |  | UNK | 0.867 | 0.904 |
| AAEL004447 | hypothetical protein |  | UNK | 0.849 | 0.993 |
| AAEL004149 | hypothetical protein |  | UNK | 0.938 | 1.176 |
| AAEL011064 | hypothetical protein |  | UNK | 1.371 | 0.869 |
| AAEL006035 | hypothetical protein |  | UNK | 1.34 | 0.949 |
| AAEL011525 | hypothetical protein |  | UNK | 1.3 | 0.869 |
| AAEL010758 | hypothetical protein |  | UNK | 1.988 |  |
| AAEL002757 | hypothetical protein |  | UNK | 1.973 |  |
| AAEL000587 | hypothetical protein |  | UNK | 1.785 |  |
| AAEL009776 | conserved hypothetical protein |  | UNK | 1.699 |  |
| AAEL002835 | conserved hypothetical protein |  | UNK | 1.653 |  |
| AAEL006046 | hypothetical protein |  | UNK | 1.617 |  |
| AAEL014693 | conserved hypothetical protein |  | UNK | 1.534 |  |
| AAEL012203 | conserved hypothetical protein |  | UNK | 1.511 |  |
| AAEL005867 | conserved hypothetical protein |  | UNK | 1.499 |  |
| AAEL007539 | hypothetical protein |  | UNK | 1.485 |  |
| AAEL001409 | conserved hypothetical protein |  | UNK | 1.434 |  |
| AAEL002963 | conserved hypothetical protein |  | UNK | 1.428 |  |
| AAEL010308 | hypothetical protein |  | UNK | 1.349 |  |
| AAEL010652 | hypothetical protein |  | UNK | 1.325 |  |
| AAEL009386 | hypothetical protein |  | UNK | 1.31 |  |
| AAEL011153 | hypothetical protein |  | UNK | 1.307 |  |
| AAEL009639 | conserved hypothetical protein |  | UNK | 1.296 |  |
| AAEL004354 | hypothetical protein |  | UNK | 1.289 |  |
| AAEL006863 | hypothetical protein |  | UNK | 1.276 |  |
| AAEL001786 | hypothetical protein |  | UNK | 1.269 |  |
| AAEL007606 | hypothetical protein |  | UNK | 1.266 |  |
| AAEL007242 | conserved hypothetical protein |  | UNK | 1.26 |  |
| AAEL008054 | conserved hypothetical protein |  | UNK | 1.259 |  |
| AAEL014415 | conserved hypothetical protein |  | UNK | 1.256 |  |
| AAEL006263 | conserved hypothetical protein |  | UNK | 1.251 |  |
| AAEL011703 | conserved hypothetical protein |  | UNK | 1.225 |  |
| AAEL002169 | conserved hypothetical protein |  | UNK | 1.22 |  |
| AAEL002168 | conserved hypothetical protein |  | UNK | 1.214 |  |
| AAEL010445 | hypothetical protein |  | UNK | 1.176 |  |
| AAEL001529 | hypothetical protein |  | UNK | 1.153 |  |
| AAEL014213 | hypothetical protein |  | UNK | 1.146 |  |
| AAEL004583 | conserved hypothetical protein |  | UNK | 1.145 |  |
| AAEL003373 | hypothetical protein |  | UNK | 1.13 |  |
| AAEL005843 | conserved hypothetical protein |  | UNK | 1.13 |  |
| AAEL002659 | hypothetical protein |  | UNK | 1.049 |  |
| AAEL012302 | conserved hypothetical protein |  | UNK | 1.045 |  |
| AAEL012293 | conserved hypothetical protein |  | UNK | 1.042 |  |
| AAEL007817 | hypothetical protein |  | UNK | 1.029 |  |
| AAEL002327 | hypothetical protein |  | UNK | 1.028 |  |
| AAEL010015 | hypothetical protein |  | UNK | 1.014 |  |
| AAEL013317 | hypothetical protein |  | UNK | 1.013 |  |
| AAEL004800 | hypothetical protein |  | UNK | 0.975 |  |
| AAEL002923 | hypothetical protein |  | UNK | 0.975 |  |
| AAEL013800 | conserved hypothetical protein |  | UNK | 0.951 |  |
| AAEL007454 | conserved hypothetical protein |  | UNK | 0.95 |  |
| AAEL001581 | conserved hypothetical protein |  | UNK | 0.948 |  |
| AAEL001376 | hypothetical protein |  | UNK | 0.945 |  |
| AAEL004854 | conserved hypothetical protein |  | UNK | 0.944 |  |
| AAEL007015 | conserved hypothetical protein |  | UNK | 0.939 |  |
| AAEL000258 | conserved hypothetical protein |  | UNK | 0.934 |  |
| AAEL002543 | conserved hypothetical protein |  | UNK | 0.93 |  |
| AAEL006520 | hypothetical protein |  | UNK | 0.921 |  |
| AAEL006275 | conserved hypothetical protein |  | UNK | 0.896 |  |
| AAEL014294 | conserved hypothetical protein |  | UNK | 0.89 |  |
| AAEL014022 | conserved hypothetical protein |  | UNK | 0.884 |  |
| AAEL004832 | conserved hypothetical protein |  | UNK | 0.879 |  |
| AAEL000316 | hypothetical protein |  | UNK | 0.873 |  |
| AAEL012754 | hypothetical protein |  | UNK | 0.868 |  |
| AAEL004408 | hypothetical protein |  | UNK | 0.865 |  |
| AAEL005007 | hypothetical protein |  | UNK | 0.862 |  |
| AAEL009163 | conserved hypothetical protein |  | UNK | 0.86 |  |
| AAEL001495 | conserved hypothetical protein |  | UNK | 0.856 |  |
| AAEL004934 | hypothetical protein |  | UNK | 0.855 |  |
| AAEL007071 | conserved hypothetical protein |  | UNK | 0.844 |  |
| AAEL004363 | conserved hypothetical protein |  | UNK | 0.836 |  |
| AAEL010034 | hypothetical protein |  | UNK | 0.835 |  |
| AAEL007433 | conserved hypothetical protein |  | UNK | 0.834 |  |
| AAEL010025 | conserved hypothetical protein |  | UNK | 0.833 |  |
| AAEL002984 | hypothetical protein |  | UNK | 0.827 |  |
| AAEL003126 | conserved hypothetical protein |  | UNK | 0.819 |  |
| AAEL011224 | hypothetical protein |  | UNK | 0.812 |  |
| AAEL008154 | hypothetical protein |  | UNK | 0.811 |  |
| AAEL000649 | conserved hypothetical protein |  | UNK | 0.804 |  |
| AAEL005755 | hypothetical protein |  | UNK | 0.803 |  |
| AAEL013724 | conserved hypothetical protein |  | UNK | 0.784 |  |
| AAEL012854 | hypothetical protein |  | UNK | 0.782 |  |
| AAEL012858 | hypothetical protein |  | UNK |  | 2.571 |
| AAEL014950 | conserved hypothetical protein |  | UNK |  | 1.671 |
| AAEL015112 | hypothetical protein |  | UNK |  | 1.571 |
| AAEL011066 | hypothetical protein |  | UNK |  | 1.521 |
| AAEL009896 | hypothetical protein |  | UNK |  | 1.466 |
| AAEL001727 | hypothetical protein |  | UNK |  | 1.435 |
| AAEL001921 | hypothetical protein |  | UNK |  | 1.373 |
| AAEL012396 | conserved hypothetical protein |  | UNK |  | 1.348 |
| AAEL005233 | hypothetical protein |  | UNK |  | 1.332 |
| AAEL015446 | conserved hypothetical protein |  | UNK |  | 1.316 |
| AAEL013708 | conserved hypothetical protein |  | UNK |  | 1.26 |
| AAEL007550 | conserved hypothetical protein |  | UNK |  | 1.246 |
| AAEL011886 | hypothetical protein |  | UNK |  | 1.222 |
| AAEL006761 | hypothetical protein |  | UNK |  | 1.22 |
| AAEL003778 | conserved hypothetical protein |  | UNK |  | 1.181 |
| AAEL002931 | hypothetical protein |  | UNK |  | 1.164 |
| AAEL010325 | hypothetical protein |  | UNK |  | 1.152 |
| AAEL013303 | conserved hypothetical protein |  | UNK |  | 1.134 |
| AAEL007414 | conserved hypothetical protein |  | UNK |  | 1.092 |
| AAEL011100 | hypothetical protein |  | UNK |  | 1.064 |
| AAEL014043 | hypothetical protein |  | UNK |  | 1.064 |
| AAEL003693 | hypothetical protein |  | UNK |  | 1.058 |
| AAEL005280 | hypothetical protein |  | UNK |  | 1.055 |
| AAEL010150 | conserved hypothetical protein |  | UNK |  | 1.053 |
| AAEL004498 | hypothetical protein |  | UNK |  | 1.025 |
| AAEL009444 | hypothetical protein |  | UNK |  | 0.992 |
| AAEL011598 | hypothetical protein |  | UNK |  | 0.987 |
| AAEL003798 | hypothetical protein |  | UNK |  | 0.984 |
| AAEL010746 | hypothetical protein |  | UNK |  | 0.982 |
| AAEL011266 | hypothetical protein |  | UNK |  | 0.976 |
| AAEL001271 | conserved hypothetical protein |  | UNK |  | 0.966 |
| AAEL002421 | hypothetical protein |  | UNK |  | 0.96 |
| AAEL005193 | hypothetical protein |  | UNK |  | 0.94 |
| AAEL007805 | hypothetical protein |  | UNK |  | 0.931 |
| AAEL013304 | conserved hypothetical protein |  | UNK |  | 0.918 |
| AAEL008142 | hypothetical protein |  | UNK |  | 0.896 |
| AAEL009570 | hypothetical protein |  | UNK |  | 0.883 |
| AAEL009322 | hypothetical protein |  | UNK |  | 0.851 |
| AAEL005236 | hypothetical protein |  | UNK |  | 0.846 |
| AAEL004018 | conserved hypothetical protein |  | UNK |  | 0.84 |
| AAEL006606 | hypothetical protein |  | UNK |  | 0.812 |
| AAEL007437 | conserved hypothetical protein |  | UNK |  | 0.799 |
| AAEL013684 | conserved hypothetical protein |  | UNK |  | 0.794 |
| AAEL007751 | predicted protein |  | UNK |  | 0.793 |
| AAEL012815 | hypothetical protein |  | UNK |  | 0.78 |
| AAEL005623 | hypothetical protein |  | UNK |  | 0.775 |
| AAEL006896 | hypothetical protein |  | UNK | -1.588 | -1.397 |
| AAEL003190 | hypothetical protein |  | UNK | -0.85 | -1.084 |
| AAEL007886 | hypothetical protein |  | UNK | -0.797 | -1.071 |
| AAEL004943 | conserved hypothetical protein |  | UNK | -1.435 | -1.034 |
| AAEL004561 | conserved hypothetical protein |  | UNK | -0.865 | -1.023 |
| AAEL005264 | hypothetical protein |  | UNK | -0.896 | -1.01 |
| AAEL011330 | conserved hypothetical protein |  | UNK | -1.195 | -0.986 |
| AAEL000186 | conserved hypothetical protein |  | UNK | -0.858 | -0.974 |
| AAEL012931 | conserved hypothetical protein |  | UNK | -1.159 | -0.922 |
| AAEL000561 | hypothetical protein |  | UNK | -0.847 | -0.917 |
| AAEL002921 | conserved hypothetical protein |  | UNK | -1.113 | -0.902 |
| AAEL001162 | conserved hypothetical protein |  | UNK | -1.013 | -0.894 |
| AAEL012361 | conserved hypothetical protein |  | UNK | -1.081 | -0.874 |
| AAEL013426 | hypothetical protein |  | UNK | -0.957 | -0.866 |
| AAEL013935 | conserved hypothetical protein |  | UNK | -0.932 | -0.853 |
| AAEL003264 | conserved hypothetical protein |  | UNK | -0.945 | -0.811 |
| AAEL003681 | hypothetical protein |  | UNK | -0.796 | -0.794 |
| AAEL005972 | hypothetical protein |  | UNK | -0.87 | -0.791 |
| AAEL008680 | conserved hypothetical protein |  | UNK | -0.896 | -0.784 |
| AAEL003088 | hypothetical protein |  | UNK | -1.215 |  |
| AAEL009270 | hypothetical protein |  | UNK | -1.145 |  |
| AAEL012878 | hypothetical protein |  | UNK | -1.08 |  |
| AAEL013895 | conserved hypothetical protein |  | UNK | -1.059 |  |
| AAEL003816 | hypothetical protein |  | UNK | -1.038 |  |
| AAEL011636 | hypothetical protein |  | UNK | -1.033 |  |
| AAEL004775 | conserved hypothetical protein |  | UNK | -1.003 |  |
| AAEL006225 | conserved hypothetical protein |  | UNK | -0.989 |  |
| AAEL009892 | conserved hypothetical protein |  | UNK | -0.961 |  |
| AAEL015457 | conserved hypothetical protein |  | UNK | -0.952 |  |
| AAEL011640 | hypothetical protein |  | UNK | -0.944 |  |
| AAEL009767 | conserved hypothetical protein |  | UNK | -0.935 |  |
| AAEL003113 | conserved hypothetical protein |  | UNK | -0.935 |  |
| AAEL008557 | conserved hypothetical protein |  | UNK | -0.928 |  |
| AAEL002856 | conserved hypothetical protein |  | UNK | -0.911 |  |
| AAEL004250 | conserved hypothetical protein |  | UNK | -0.9 |  |
| AAEL003451 | conserved hypothetical protein |  | UNK | -0.883 |  |
| AAEL010249 | conserved hypothetical protein |  | UNK | -0.872 |  |
| AAEL014937 | hypothetical protein |  | UNK | -0.871 |  |
| AAEL004552 | conserved hypothetical protein |  | UNK | -0.861 |  |
| AAEL005000 | conserved hypothetical protein |  | UNK | -0.858 |  |
| AAEL004809 | conserved hypothetical protein |  | UNK | -0.851 |  |
| AAEL010768 | conserved hypothetical protein |  | UNK | -0.833 |  |
| AAEL004960 | hypothetical protein |  | UNK | -0.824 |  |
| AAEL003822 | conserved hypothetical protein |  | UNK | -0.821 |  |
| AAEL004473 | conserved hypothetical protein |  | UNK | -0.808 |  |
| AAEL009952 | hypothetical protein |  | UNK | -0.806 |  |
| AAEL002109 | conserved hypothetical protein |  | UNK | -0.802 |  |
| AAEL007849 | conserved hypothetical protein |  | UNK | -0.791 |  |
| AAEL010507 | hypothetical protein |  | UNK | -0.791 |  |
| AAEL015340 | hypothetical protein |  | UNK | -0.779 |  |
| AAEL013725 | conserved hypothetical protein |  | UNK |  | -1.272 |
| AAEL000526 | conserved hypothetical protein |  | UNK |  | -0.904 |
| AAEL010770 | hypothetical protein |  | UNK |  | -0.869 |
| AAEL013988 | conserved hypothetical protein |  | UNK |  | -0.847 |
| AAEL015507 | conserved hypothetical protein |  | UNK |  | -0.853 |
| AAEL001573 | conserved hypothetical protein |  | UNK |  | -1.133 |
| AAEL007045 | conserved hypothetical protein |  | UNK |  | -1.018 |
| AAEL008403 | conserved hypothetical protein |  | UNK |  | -0.981 |
| AAEL007859 | conserved hypothetical protein |  | UNK |  | -0.955 |
| AAEL011635 | conserved hypothetical protein |  | UNK |  | -0.948 |
| AAEL008059 | conserved hypothetical protein |  | UNK |  | -0.938 |
| AAEL014633 | conserved hypothetical protein |  | UNK |  | -0.916 |
| AAEL011119 | hypothetical protein |  | UNK |  | -0.915 |
| AAEL002431 | conserved hypothetical protein |  | UNK |  | -0.857 |
| AAEL005640 | conserved hypothetical protein |  | UNK |  | -0.826 |
| AAEL013740 | hypothetical protein |  | UNK |  | -0.823 |
| AAEL009440 | conserved hypothetical protein |  | UNK |  | -0.799 |
| AAEL002087 | conserved hypothetical protein |  | UNK |  | -0.783 |
| AAEL008436 | conserved hypothetical protein |  | UNK |  | -0.779 |
