# Supplementary material for: Dengue Virus Inhibits Immune Responses in Aedes aegypti Cells
Source: PLoS One. 2010 May 18;5(5):e10678. doi: 10.1371/journal.pone.0010678 (PMC2872661; doi:10.1371/journal.pone.0010678)
Supplement: Table S3 — Averaged data from three biological replicate semi-quantitative PCR assays. Averaged data from three biological replicate semi-quantitative PCR assays of cecropin and defensin expression at varying time points post-secondary bacterial challenge of DENV- or mock-infected Aag2 cells. The fold change in gene expression compared to the 0 h time point is shown. p-values are for a Student's t-test comparing fold change in gene expression upon secondary bacterial challenge in DENV- and mock-infected cells. *, p<0.05; SEM, standard error of the mean; ND, non-detectable. (0.05 MB DOC) [file pone.0010678.s003.doc]

**Table S3:** Averaged data from three biological replicate semi-quantitative PCR assays of cecropin and defensin expression at varying time points post-secondary bacterial challenge of DENV- or mock-infected Aag2 cells. The fold change in gene expression compared to the 0 h time point is shown. p-values are for a Student’s t-test comparing fold change in gene expression upon secondary bacterial challenge in DENV- and mock-infected cells. *, p < 0.05; SEM, standard error of the mean; ND, non-detectable.

| **Hours post-secondary bacterial challenge** | **Sample** | **CEC G (AAEL015515-RA)** | | | **DEF C (AAEL003832-RA)** | | |
| --- | --- | --- | --- | --- | --- | --- | --- |
|  |  | **Fold change** | **SEM** | **p-value** | **Fold change** | **SEM** | **p-value** |
| **2** | DENV / *E. coli* | 2.19 | 0.40 | 0.011* | 1.35 | 0.21 | 0.003* |
|  | - / *E. coli* | 4.44 | 0.30 | - | 2.76 | 0.07 | - |
|  | DENV / *S. aureus* | 1.36 | 0.08 | 0.793 | 1.98 | 0.17 | 0.14 |
|  | - / *S. aureus* | 1.43 | 0.23 | - | 2.33 | 0.07 | - |
|  | DENV / - | 0.13 | 0.13 | 0.379 | 1.41 | 0.09 | 0.19 |
|  | - / - | ND | - | - | 1.65 | 0.12 | - |
| **6** | DENV / *E. coli* | 2.63 | 0.23 | 0.11 | 2.48 | 0.17 | 0.23 |
|  | - / *E. coli* | 4.17 | 0.73 | - | 3.05 | 0.37 | - |
|  | DENV / *S. aureus* | 1.49 | 0.20 | 0.01* | 2.38 | 0.05 | 0.65 |
|  | - / *S. aureus* | 3.02 | 0.30 | - | 2.69 | 0.63 | - |
|  | DENV / - | 0.25 | 0.13 | 0.23 | 2.28 | 0.13 | 0.63 |
|  | - / - | 0.46 | 0.07 | - | 2.36 | 0.08 | - |
| **18** | DENV / *E. coli* | 2.59 | 0.24 | 0.17 | 3.39 | 0.06 | 0.004* |
|  | - / *E. coli* | 3.23 | 0.30 | - | 4.95 | 0.25 | - |
|  | DENV / *S. aureus* | 2.38 | 0.38 | 0.14 | 3.77 | 0.33 | 0.04* |
|  | - / *S. aureus* | 4.01 | 0.79 | - | 5.71 | 0.56 | - |
|  | DENV / - | 0.17 | 0.09 | 0.28 | 1.85 | 0.06 | 0.58 |
|  | - / - | 0.05 | 0.05 | - | 2.01 | 0.27 | - |
