# Supplementary material for: Dengue Virus Inhibits Immune Responses in Aedes aegypti Cells
Source: PLoS One. 2010 May 18;5(5):e10678. doi: 10.1371/journal.pone.0010678 (PMC2872661; doi:10.1371/journal.pone.0010678)
Supplement: Table S4 — Average of four biological replicate OD595 readings. Average of four biological replicate OD595 readings for (A) E. coli, (B) S. aureus, and (C) M. luteus after a 12-h incubation at 28°C with DENV- or mock-infected Aag2 cells. p-values are for a Student's t-test comparing OD595 of bacteria incubated with DENV- and mock-infected cells. *, p<0.05; SEM, standard error of the mean. (0.06 MB DOC) [file pone.0010678.s004.doc]

**Table S4:** Average of four biological replicate OD595 readings for (A) *E. coli,* (B) *S. aureus,* and (C) *M. luteus* after a 12-h incubation at 28°C with DENV- or mock-infected Aag2 cells. p-values are for a Student’s t-test comparing OD595 of bacteria incubated with DENV- and mock-infected cells. *, p < 0.05; SEM, standard error of the mean.

***A) E. coli***

| **- log_10_ dilution** | **Aag2 / DENV / *E. coli*** | | | **Aag2 / *E. coli*** | | ***E. coli*** | |
| --- | --- | --- | --- | --- | --- | --- | --- |
|  | **Mean OD595** | **SEM** | **p-value** | **Mean OD595** | **SEM** | **Mean OD595** | **SEM** |
| **1** | 0.351 | 0.015 | 0.575 | 0.362 | 0.011 | 0.335 | 0.025 |
| **2** | 0.260 | 0.002 | 0.418 | 0.252 | 0.009 | 0.263 | 0.005 |
| **3** | 0.217 | 0.001 | 0.127 | 0.205 | 0.007 | 0.227 | 0.002 |
| **4** | 0.184 | 0.004 | 0.016* | 0.148 | 0.010 | 0.192 | 0.004 |
| **5** | 0.138 | 0.004 | 0.010* | 0.106 | 0.007 | 0.164 | 0.007 |
| **6** | 0.103 | 0.005 | 0.015* | 0.085 | 0.003 | 0.119 | 0.011 |
| **7** | 0.084 | 0.005 | 0.027* | 0.068 | 0.001 | 0.076 | 0.005 |
| **8** | 0.072 | 0.005 | 0.173 | 0.062 | 0.003 | 0.070 | 0.002 |

***B) S. aureus***

| **- log_10_ dilution** | **Aag2 / DENV / *S. aureus*** | | | **Aag2 / *S. aureus*** | | ***S. aureus*** | |
| --- | --- | --- | --- | --- | --- | --- | --- |
|  | **Mean OD595** | **SEM** | **p-value** | **Mean OD595** | **SEM** | **Mean OD595** | **SEM** |
| **1** | 0.557 | 0.003 | 0.348 | 0.563 | 0.010 | 0.520 | 0.007 |
| **2** | 0.548 | 0.004 | 0.165 | 0.536 | 0.013 | 0.506 | 0.017 |
| **3** | 0.514 | 0.010 | 0.220 | 0.499 | 0.006 | 0.486 | 0.027 |
| **4** | 0.435 | 0.011 | 0.297 | 0.421 | 0.007 | 0.419 | 0.023 |
| **5** | 0.334 | 0.004 | 0.929 | 0.335 | 0.014 | 0.348 | 0.021 |
| **6** | 0.287 | 0.024 | 0.369 | 0.263 | 0.008 | 0.288 | 0.024 |
| **7** | 0.251 | 0.019 | 0.343 | 0.227 | 0.027 | 0.245 | 0.036 |
| **8** | 0.178 | 0.016 | 0.577 | 0.190 | 0.023 | 0.193 | 0.031 |

***C) M. luteus***

| **- log_10_ dilution** | **Aag2 / DENV / *M. luteus*** | | | **Aag2 / *M. luteus*** | | ***M. luteus*** | |
| --- | --- | --- | --- | --- | --- | --- | --- |
|  | **Mean OD595** | **SEM** | **p-value** | **Mean OD595** | **SEM** | **Mean OD595** | **SEM** |
| **1** | 0.344 | 0.014 | 0.007 | 0.362 | 0.013 | 0.346 | 0.010 |
| **2** | 0.203 | 0.010 | 0.005 | 0.196 | 0.006 | 0.204 | 0.021 |
| **3** | 0.118 | 0.012 | 0.006 | 0.121 | 0.003 | 0.129 | 0.011 |
| **4** | 0.075 | 0.008 | 0.004 | 0.078 | 0.005 | 0.088 | 0.005 |
| **5** | 0.056 | 0.001 | 0.001 | 0.056 | 0.004 | 0.054 | 0.002 |
| **6** | 0.052 | 0.004 | 0.002 | 0.048 | 0.002 | 0.045 | 0.004 |
| **7** | 0.047 | 0.002 | 0.001 | 0.049 | 0.003 | 0.041 | 0.001 |
| **8** | 0.048 | 0.002 | 0.001 | 0.047 | 0.003 | 0.039 | 0.001 |
