# Supplementary material for: Dengue Virus Inhibits Immune Responses in Aedes aegypti Cells
Source: PLoS One. 2010 May 18;5(5):e10678. doi: 10.1371/journal.pone.0010678 (PMC2872661; doi:10.1371/journal.pone.0010678)
Supplement: Table S5 — Averaged data from three biological replicate plaque assays of DENV titers. Averaged data from three biological replicate plaque assays of DENV titers in cell culture supernatants following pre-immune stimulation with E. coli, S. aureus, or PBS. p-values are for a Student's t-test comparing DENV titers in bacteria-stimulated cells with PBS-treated cells. *, p<0.05; SEM, standard error of the mean. (0.04 MB DOC) [file pone.0010678.s005.doc]

**Table S5:** Averaged data from three biological replicate plaque assays of DENV titers in cell culture supernatants following pre-immune stimulation with *E. coli, S. aureus,* or PBS. p-values are for a Student’s t-test comparing DENV titers in bacteria-stimulated cells with PBS-treated cells. *, p < 0.05; SEM, standard error of the mean.

| **Days post-DENV infection** | ***E. coli*** | | | ***S. aureus*** | | | **PBS** | |
| --- | --- | --- | --- | --- | --- | --- | --- | --- |
|  | **Mean PFU / ml** | **SEM** | **p-value** | **Mean PFU / ml** | **SEM** | **p-value** | **Mean PFU / ml** | **SEM** |
| **1** | 5.33 x 10^1^ | 2.33 x 10^1^ | 0.15 | 6.67 | 6.67 | 0.72 | 1.00 x 10^1^ | 5.77 |
| **2** | 1.61 x 10^3^ | 6.77 x 10^1^ | 0.0003* | 1.36 x 10^3^ | 9.21 x 10^1^ | 0.005* | 8.23 x 10^2^ | 1.33 x 10^1^ |
| **3** | 2.14 x 10^4^ | 4.03 x 10^3^ | 0.08 | 7.52 x 10^3^ | 2.16 x 10^3^ | 0.20 | 1.14 x 10^4^ | 1.41 x 10^3^ |
| **4** | 2.70 x 10^5^ | 5.11 x 10^4^ | 0.03* | 7.43 x 10^4^ | 2.21 x 10^4^ | 0.47 | 9.23 x 10^4^ | 5.67 x 10^3^ |
| **5** | 7.93 x 10^5^ | 2.03 x 10^5^ | 0.14 | 2.93 x 10^5^ | 3.77 x 10^4^ | 0.08 | 4.12 x 10^5^ | 3.56 x 10^4^ |
| **6** | 7.18 x 10^5^ | 3.66 x 10^4^ | 0.20 | 5.67 x 10^5^ | 8.92 x 10^4^ | 0.84 | 5.37 x 10^5^ | 1.13 x 10^5^ |
| **7** | 1.00 x 10^6^ | 3.08 x 10^5^ | 0.37 | 8.28 x 10^5^ | 2.07 x 10^5^ | 0.55 | 6.65 x 10^5^ | 1.41 x 10^5^ |
