# Supplementary material for: Dengue Virus Inhibits Immune Responses in Aedes aegypti Cells
Source: PLoS One. 2010 May 18;5(5):e10678. doi: 10.1371/journal.pone.0010678 (PMC2872661; doi:10.1371/journal.pone.0010678)
Supplement: Table S6 — Cluster analysis. Cluster analysis of 238 genes that were regulated in at least two of three treatments: DENV infection in the cell line, HIA DENV infection in the cell line, Cactus silencing in A. aegypti mosquitoes (Figure 1D). (0.36 MB DOC) [file pone.0010678.s006.doc]

**Table S6:** Cluster analysis of 238 genes that were regulated in at least two of three treatments: DENV infection in the cell line, HIA DENV infection in the cell line, Cactus silencing in *A. aegypti* mosquitoes (Figure 1D).

| **GENE ID** | **NAME** | **Functional group** | **Log_2_ fold** | | | **Cluster** |
| --- | --- | --- | --- | --- | --- | --- |
|  |  |  | **DENV** | **HIA DENV** | **Cactus** |  |
| AAEL014353 | conserved hypothetical protein | PROT | 0.98 | 1.088 | 1.372 | I |
| AAEL007363 | leucinech transmembrane protein | I | 1.055 | 0.837 | 0.993 | I |
| AAEL002688 | glucosyl/glucuronosyl transferases | M | 0.905 | 0.862 | 0.807 | I |
| AAEL011330 | conserved hypothetical protein | U | -1.195 | -0.986 | -2.089 | I |
| AAEL011596 | mitotic checkpoint serine/threonine-protein kinase bub1 and bubr1 | D | -1.062 | -0.778 | -1.989 | I |
| AAEL001703 | serine-type enodpeptidase, putative | DIG | -1.281 | -1.12 | -1.856 | I |
| AAEL012187 | lethal(3)malignant brain tumor | D | -1.001 | -0.863 | -1.84 | I |
| AAEL011220 | Ati or CPXV158 protein, putative | D | -1.407 | -1.087 | -1.813 | I |
| AAEL002921 | conserved hypothetical protein | U | -1.113 | -0.902 | -1.797 | I |
| AAEL001635 | conserved hypothetical protein | D | -0.995 | -0.989 | -1.73 | I |
| AAEL004139 | hypothetical protein | D | -0.844 | -0.989 | -1.618 | I |
| AAEL007046 | mitochondrial brown fat uncoupling protein | RSM | -1.08 | -1.452 | -1.604 | I |
| AAEL002569 | serine/threonine kinase | D | -0.893 | -0.803 | -1.602 | I |
| AAEL003681 | hypothetical protein | U | -0.796 | -0.794 | -1.592 | I |
| AAEL013426 | hypothetical protein | U | -0.957 | -0.866 | -1.565 | I |
| AAEL012418 | deoxyribonuclease ii | M | -1.046 | -0.963 | -1.257 | I |
| AAEL005216 | conserved hypothetical protein | D | -1.133 | -0.909 | -1.158 | I |
| AAEL006896 | hypothetical protein | U | -1.588 | -1.397 | -1.109 | I |
| AAEL011009 | fibrinogen and fibronectin | I | -1.096 | -0.899 | -1.081 | I |
| Aaeg:N44512 | DCE | I | -2.526 | -1.864 | -1.078 | I |
| AAEL004943 | conserved hypothetical protein | U | -1.435 | -1.034 | -0.947 | I |
| AAEL006179 | tubulin alpha chain | CS | -1.537 | -1.116 | -0.906 | I |
| AAEL000186 | conserved hypothetical protein | U | -0.858 | -0.974 | -0.899 | I |
| AAEL014169 | small nuclear ribonucleoprotein, core, putative | RTT | -1.255 | -0.814 | -0.892 | I |
| AAEL000703 | glycogen phosphorylase | D | -0.821 | -1.233 | -0.878 | I |
| AAEL012361 | conserved hypothetical protein | U | -1.081 | -0.874 | -0.866 | I |
| AAEL004848 | small nuclear ribonucleoprotein, core, putative | RTT | -1.274 | -0.894 | -0.822 | I |
| AAEL013453 | sarcolemmal associated protein, putative | D | -0.792 | -0.813 | -0.816 | I |
| Aaeg:N49982 | CLIPB16 | I | 0.778 | 1.526 | 0.049 | II |
| AAEL011552 | hypothetical protein | D | 0.889 | 1.342 | 0.205 | II |
| AAEL014246 | glucosyl/glucuronosyl transferases | M | 1.062 | 1.31 | -0.536 | II |
| AAEL004149 | hypothetical protein | U | 0.938 | 1.176 | 0.134 | II |
| AAEL004197 | hypothetical protein | D | 1.731 | 1.116 | -0.661 | II |
| AAEL000820 | dimethylaniline monooxygenase | D | 1.226 | 1.107 | -0.124 | II |
| AAEL011384 | hypothetical protein | D | 1.042 | 1.028 | -0.746 | II |
| AAEL004447 | hypothetical protein | U | 0.849 | 0.993 | -0.023 | II |
| AAEL003989 | GTP-binding protein alpha subunit, gna | D | 1.313 | 0.979 | 0.095 | II |
| AAEL003223 | hypothetical protein | U | 1.832 | 0.972 | -0.429 | II |
| AAEL006035 | hypothetical protein | U | 1.34 | 0.949 | -0.352 | II |
| AAEL007898 | calmin | CS | 0.838 | 0.934 | -0.038 | II |
| AAEL002470 | hypothetical protein | U | 0.867 | 0.904 | 0.421 | II |
| AAEL014301 | hypothetical protein | D | 0.791 | 0.899 | 0.166 | II |
| AAEL006619 | conserved hypothetical protein | D | 1.643 | 0.896 | 0.216 | II |
| AAEL010951 | glutamate decarboxylase | I | 0.814 | 0.894 | 0.009 | II |
| AAEL011064 | hypothetical protein | U | 1.371 | 0.869 | -0.016 | II |
| AAEL011525 | hypothetical protein | U | 1.3 | 0.869 | 0.235 | II |
| AAEL010674 | hypothetical protein | D | 1.068 | 0.842 | -0.584 | II |
| AAEL003220 | rho-type gtpase activating protein | D | 1.102 | 0.828 | -0.368 | II |
| AAEL010301 | conserved hypothetical protein | D | 0.821 | 0.81 | -0.74 | II |
| AAEL001313 | conserved hypothetical protein | U | 0.965 | 0.805 | -0.29 | II |
| AAEL007401 | roundabout, putative | D | 1.358 | 0.803 | 0.257 | II |
| AAEL011105 | adducin | D | 0.864 | 0.797 | -0.351 | II |
| AAEL013882 | tkr | D | 1.481 | 0.796 | 0.176 | II |
| AAEL013028 | zinc finger protein | D | 1.065 | 0.794 | 0.392 | II |
| AAEL010989 | hypothetical protein | RSM | 1.511 | 0.79 | 0.351 | II |
| AAEL009377 | RAS protein, putative | D | 0.888 | 0.786 | -0.669 | II |
| AAEL014991 | hypothetical protein | D | 0.877 | 0.786 | -0.42 | II |
| AAEL004715 | b-cell translocation protein | D | 1.534 | 0.767 | 0.84 | II |
| AAEL002899 | hypothetical protein | D | 1.473 | 0.518 | 1.348 | II |
| AAEL002963 | conserved hypothetical protein | U | 1.428 | 0.695 | 1.182 | II |
| AAEL004913 | adpbosylation factor, arf | D | 1.317 | 0.559 | 0.79 | II |
| AAEL007613 | toll | I | 1.179 | 0.561 | 1.307 | II |
| AAEL005763 | lysosomal alpha-mannosidase (mannosidase alpha class 2b member 1) | M | 0.962 | 0.454 | 0.985 | II |
| AAEL005861 | vacuolar sorting protein (vps) | D | 0.878 |  | 1.021 | II |
| Aaeg:N51900 | HSC70-3 | I | 0.789 | 0.3 | 0.964 | II |
| AAEL003965 | calpain, putative | PROT | 0.789 |  | 1.693 | II |
| AAEL003593 | hypothetical protein | CSR |  | 1.295 | 2.718 | II |
| AAEL011455 | galactose-specific C-type lectin, putative | I | 0.171 | 1.622 | 2.473 | II |
| AAEL011598 | hypothetical protein | U | 0.45 | 0.987 | 2.054 | II |
| AAEL014350 | hypothetical protein | PROT |  | 1.501 | 1.604 | II |
| Aaeg:N18089 | DCE | I |  | 1.409 | 1.366 | II |
| AAEL003508 | serine-pyruvate aminotransferase | D |  | 0.864 | 1.308 | II |
| Aaeg:N32065 | CTL | I |  | 1.315 | 1.197 | II |
| AAEL011619 | galactose-specific C-type lectin, putative | I | -0.199 | 1.238 | 1.129 | II |
| AAEL007696 | embryonic polarity dorsal | I | 0.225 | 0.807 | 1.005 | II |
| AAEL011623 | conserved hypothetical protein | D | 0.422 | 1.18 | 0.849 | II |
| AAEL013525 | Timp-3, putative | M |  | 0.848 | 0.818 | II |
| AAEL007539 | hypothetical protein | U | 1.485 |  | -2.16 | III |
| AAEL005843 | conserved hypothetical protein | U | 1.13 |  | -1.804 | III |
| AAEL014608 | cytochrome P450 | RSM | 0.935 | 0.589 | -1.794 | III |
| AAEL013510 | smaug protein | D | 0.848 |  | -1.541 | III |
| AAEL002551 | DNA topoisomerase type I | RTT | 0.859 |  | -1.406 | III |
| AAEL000857 | conserved hypothetical protein | D | 0.801 |  | -1.324 | III |
| AAEL002251 | conserved hypothetical protein | D | 0.869 |  | -1.314 | III |
| AAEL007705 | hect E3 ubiquitin ligase | D | 1.222 | 0.494 | -1.311 | III |
| AAEL003540 | conserved hypothetical protein | D | 0.91 | 0.32 | -1.302 | III |
| AAEL002166 | leucine rich repeat (in flii) interacting protein | I | 1.131 | 0.3 | -1.294 | III |
| AAEL002771 | microtubule binding protein, putative | CS | 0.912 |  | -1.278 | III |
| AAEL005755 | hypothetical protein | U | 0.803 | 0.774 | -1.158 | III |
| AAEL006651 | dystrophin | D | 0.86 | 0.52 | -1.097 | III |
| AAEL000430 | hypothetical protein | D | 1.158 | 0.417 | -1.093 | III |
| AAEL008171 | double-stranded RNA-binding protein zn72d | D | 1.163 | 0.282 | -1.093 | III |
| AAEL013317 | hypothetical protein | U | 1.013 | 0.373 | -1.065 | III |
| AAEL002705 | nucleolar protein c7b | D | 1.211 |  | -1.039 | III |
| AAEL001375 | Y-box binding protein | D | 0.942 |  | -1.014 | III |
| AAEL000258 | conserved hypothetical protein | U | 0.934 | 0.182 | -1.005 | III |
| AAEL004722 | GABA-A receptor interacting factor-1, putative | D | 1.136 |  | -0.984 | III |
| AAEL002430 | n-acetylglucosamine-6-phosphate deacetylase | M | 1.061 | 0.715 | -0.966 | III |
| AAEL009422 | conserved hypothetical protein | D | 1.072 | 0.365 | -0.945 | III |
| AAEL001919 | protein tyrosine phosphatase, nonceptor type nt1 | D | 0.822 | 0.138 | -0.938 | III |
| AAEL013653 | tata-box binding protein | RTT | 0.987 |  | -0.897 | III |
| AAEL012584 | DNA topoisomerase/gyrase | RTT | 1.165 | 0.317 | -0.883 | III |
| AAEL010256 | E3 ubiquitin ligase | M | 1.173 |  | -0.883 | III |
| AAEL006687 | exportin | M | 1.17 |  | -0.876 | III |
| AAEL007011 | conserved hypothetical protein | D | 0.828 | 0.452 | -0.858 | III |
| AAEL000193 | histone-lysine n-methyltransferase | RTT | 0.777 |  | -0.854 | III |
| AAEL007242 | conserved hypothetical protein | U | 1.26 | 0.571 | -0.81 | III |
| AAEL000262 | conserved hypothetical protein | D | 0.922 | 0.446 | -0.805 | III |
| AAEL007653 | allantoinase | D | 1.247 | 1.294 | -1.635 | III |
| AAEL003025 | hypothetical protein | U | 1.381 | 1.018 | -1.215 | III |
| AAEL008027 | hypothetical protein | D | 1.227 | 0.789 | -1.191 | III |
| AAEL010755 | hypothetical protein | D | 1.299 | 1.012 | -1.143 | III |
| AAEL010229 | hypothetical protein | D |  | 1.068 | -2.354 | IV |
| AAEL006699 | fibrinogen and fibronectin | I |  | 1.546 | -1.297 | IV |
| AAEL009570 | hypothetical protein | U |  | 0.883 | -1.223 | IV |
| AAEL007414 | conserved hypothetical protein | U |  | 1.092 | -1.061 | IV |
| AAEL002120 | zinc finger protein | D | 0.265 | 0.837 | -0.975 | IV |
| AAEL002704 | serine protease inhibitor (serpin-4), putative | I | 0.137 | 1.149 | -0.917 | IV |
| AAEL000006 | phosphoenolpyruvate carboxykinase | M |  | 0.859 | -0.915 | IV |
| AAEL015446 | conserved hypothetical protein | U |  | 1.316 | -0.864 | IV |
| AAEL011764 | prophenoloxidase | I | -0.191 | 1.537 | -0.844 | IV |
| AAEL013417 | fibrinogen and fibronectin | I | 0.556 | 1.376 | -0.837 | IV |
| AAEL000227 | epithelial membrane protein | I | 0.62 | 0.803 | -0.835 | IV |
| AAEL009842 | keratinocyte lectin, putative | I | -0.562 | -0.957 | 2.398 | IV |
| AAEL014139 | proacrosin, putative | M |  | -0.94 | 2.341 | IV |
| AAEL000627 | antibacterial peptide, putative | I |  | -1.19 | 1.239 | IV |
| AAEL000611 | antibacterial peptide, putative | I | -1.547 | -1.065 | 1.33 | V |
| AAEL015515 | antibacterial peptide, putative | I | -1.323 | -0.942 | 1.394 | V |
| AAEL003626 | sodium/shloride dependent amino acid transporter | TRP | -1.301 | -0.896 | 1.87 | V |
| AAEL000598 | antibacterial peptide, putative | I | -1.176 | -1.144 | 0.953 | V |
| AAEL009384 | fibrinogen and fibronectin | I | -1.144 | -0.787 | 2.312 | V |
| AAEL001163 | macroglobulin/complement | I | -1.076 | -1.001 | 0.81 | V |
| AAEL003832 | conserved hypothetical protein | I | -0.992 | -1.016 | 0.95 | V |
| AAEL009474 | peptidoglycan recognition protein-lc isoform | I | -0.992 | -0.93 | 0.94 | V |
| AAEL005800 | serine protease, putative | I | -1.037 | -0.273 | 2.639 | V |
| AAEL008646 | fibrinogen and fibronectin | D | -1.036 | -0.638 | 1.642 | V |
| AAEL007107 | serine protease, putative | I | -1.027 | -0.573 | 1.586 | V |
| AAEL015457 | conserved hypothetical protein | U | -0.952 | -0.558 | 1.868 | V |
| AAEL002601 | serine protease, putative | I | -0.907 | -0.636 | 1.258 | V |
| AAEL007626 | gram-negative bacteria binding protein | I | -0.906 | -0.526 | 0.953 | V |
| AAEL003632 | clip-domain serine protease, putative | I | -0.874 | -0.671 | 2.032 | V |
| AAEL011607 | galactose-specific C-type lectin, putative | I | -0.848 | -0.355 | 1.649 | V |
| AAEL003857 | conserved hypothetical protein | I | -0.777 | -0.721 | 0.999 | V |
| AAEL013350 | heat shock protein 26kD, putative | RSM | -1.91 | -1.532 | 0.228 | VI |
| AAEL012086 | hypothetical protein | D | -1.706 | -1.378 |  | VI |
| AAEL013605 | hypothetical protein | PROT | -1.358 | -1.359 | 0.29 | VI |
| AAEL009520 | hypothetical protein | D | -1.978 | -1.272 |  | VI |
| AAEL015272 | zinc carboxypeptidase | PROT | -1.219 | -1.18 | -0.125 | VI |
| AAEL005107 | hypothetical protein | PROT | -1.403 | -1.18 | 0.403 | VI |
| AAEL007677 | phospholysine phosphohistidine inorganic pyrophosphate phosphatase | D | -0.971 | -1.121 | -0.674 | VI |
| AAEL010823 | ATP synthase delta chain | TRP | -0.969 | -1.117 | -0.629 | VI |
| AAEL006473 | arginine/serinech splicing factor | RTT | -0.908 | -1.097 | -0.47 | VI |
| AAEL003190 | hypothetical protein | U | -0.85 | -1.084 | -0.493 | VI |
| AAEL007886 | hypothetical protein | U | -0.797 | -1.071 | -0.649 | VI |
| AAEL004025 | glucose dehydrogenase | TRP | -1.051 | -1.025 | -0.118 | VI |
| AAEL004561 | conserved hypothetical protein | U | -0.865 | -1.023 | -0.459 | VI |
| AAEL005264 | hypothetical protein | U | -0.896 | -1.01 | 0.162 | VI |
| AAEL002542 | triosephosphate isomerase | M | -1.113 | -1.009 | -0.577 | VI |
| AAEL010372 | aldehyde oxidase | RSM | -1.204 | -0.993 | -0.105 | VI |
| AAEL012014 | l-lactate dehydrogenase | M | -1.069 | -0.966 | 0.63 | VI |
| AAEL013693 | excision repair cross-complementing 1 ercc1 | RSM | -0.898 | -0.954 | -0.736 | VI |
| AAEL000541 | fasciclin, putative | D | -1.096 | -0.944 | -0.76 | VI |
| AAEL012931 | conserved hypothetical protein | U | -1.159 | -0.922 | -0.583 | VI |
| AAEL000561 | hypothetical protein | U | -0.847 | -0.917 | -0.532 | VI |
| AAEL012308 | aldehyde oxidase | RSM | -1.172 | -0.912 | -0.19 | VI |
| AAEL007801 | exonuclease | RTT | -1.063 | -0.907 | -0.503 | VI |
| AAEL003195 | carboxylesterase | RSM | -0.937 | -0.906 | 0.009 | VI |
| AAEL010677 | oxidoreductase | RSM | -1.212 | -0.898 | -0.713 | VI |
| AAEL001162 | conserved hypothetical protein | U | -1.013 | -0.894 | -0.707 | VI |
| AAEL004221 | glycogen synthase | D | -0.87 | -0.893 | -0.753 | VI |
| AAEL004150 | fibrinogen and fibronectin | D | -0.907 | -0.889 |  | VI |
| AAEL010380 | aldehyde oxidase | RSM | -0.908 | -0.88 |  | VI |
| AAEL003985 | small nuclear ribonucleoprotein, core, putative | RTT | -0.783 | -0.879 | -0.35 | VI |
| AAEL013935 | conserved hypothetical protein | U | -0.932 | -0.853 | -0.742 | VI |
| AAEL003651 | conserved hypothetical protein | D | -0.91 | -0.824 | -0.347 | VI |
| AAEL010642 | poly(A)-binding protein, putative | RTT | -0.971 | -0.818 | -0.623 | VI |
| AAEL003729 | conserved hypothetical protein | D | -1.064 | -0.813 | -0.303 | VI |
| AAEL003264 | conserved hypothetical protein | U | -0.945 | -0.811 | -0.616 | VI |
| AAEL001650 | conserved hypothetical protein | D | -1.046 | -0.808 | 0.121 | VI |
| AAEL009237 | glycoside hydrolases | M | -0.856 | -0.801 | -0.49 | VI |
| AAEL005972 | hypothetical protein | U | -0.87 | -0.791 | -0.532 | VI |
| AAEL008680 | conserved hypothetical protein | U | -0.896 | -0.784 | -0.575 | VI |
| AAEL012238 | glutaredoxin, putative | D | -0.788 | -0.784 | -0.434 | VI |
| AAEL004229 | glutathione transferase AtGST, putative | D | -0.87 | -0.781 | 0.18 | VI |
| AAEL014251 | conserved hypothetical protein | I | -0.837 | -0.64 | -1.655 | VI |
| AAEL002783 | mitochondrial ribosomal protein, L37, putative | RSM | -0.796 | -0.435 | -1.638 | VI |
| AAEL005000 | conserved hypothetical protein | U | -0.858 | -0.492 | -1.633 | VI |
| AAEL015555 | conserved hypothetical protein | D | -0.978 |  | -1.563 | VI |
| AAEL002739 | conserved hypothetical protein | D | -0.879 | -0.244 | -1.54 | VI |
| AAEL007564 | zinc finger protein | D | -0.817 | -0.586 | -1.487 | VI |
| AAEL014287 | deoxycytidylate deaminase | M | -0.818 | -0.72 | -1.482 | VI |
| AAEL008557 | conserved hypothetical protein | U | -0.928 | -0.698 | -1.471 | VI |
| AAEL009270 | hypothetical protein | U | -1.145 |  | -1.463 | VI |
| AAEL013974 | conserved hypothetical protein | D | -1.106 | -0.567 | -1.425 | VI |
| AAEL011362 | hypothetical protein | D | -0.825 | -0.466 | -1.405 | VI |
| AAEL012455 | alcohol dehydrogenase | M | -0.895 | -0.641 | -1.369 | VI |
| AAEL004775 | conserved hypothetical protein | U | -1.003 | -0.523 | -1.299 | VI |
| AAEL002888 | williams-beuren syndrome critical region protein | D | -0.812 | -0.725 | -1.264 | VI |
| AAEL015658 | conserved hypothetical protein | D | -0.834 | -0.469 | -1.256 | VI |
| AAEL005557 | hypothetical protein | D | -0.916 | -0.455 | -1.175 | VI |
| AAEL008865 | oligoribonuclease, mitochondrial | M | -0.833 | -0.73 | -1.106 | VI |
| AAEL004450 | cytochrome b5, putative | RSM | -0.796 | -0.518 | -1.089 | VI |
| AAEL007355 | mitochondrial ribosomal protein, S18A, putative | RSM | -0.89 | -0.568 | -1.057 | VI |
| AAEL010013 | wdpeat protein | D | -0.881 | -0.593 | -1.05 | VI |
| AAEL011233 | SM protein G, putative | M | -1.359 | -0.583 | -1.004 | VI |
| AAEL015236 | signal recognition particle, 9kD-subunit, putative | RTT | -0.861 | -0.435 | -0.989 | VI |
| AAEL011636 | hypothetical protein | U | -1.033 | -0.556 | -0.981 | VI |
| AAEL002744 | hypothetical protein | D | -0.912 | -0.435 | -0.976 | VI |
| AAEL011640 | hypothetical protein | U | -0.944 | -0.58 | -0.969 | VI |
| AAEL001280 | 28S ribosomal protein S15, mitochondrial precursor | RTT | -0.902 | -0.427 | -0.957 | VI |
| AAEL012391 | conserved hypothetical protein | D | -1.146 | -0.533 | -0.94 | VI |
| AAEL006787 | conserved hypothetical protein | D | -0.782 | -0.358 | -0.93 | VI |
| AAEL010249 | conserved hypothetical protein | U | -0.872 | -0.273 | -0.922 | VI |
| AAEL003451 | conserved hypothetical protein | U | -0.883 |  | -0.897 | VI |
| AAEL001838 | conserved hypothetical protein | D | -0.843 | -0.51 | -0.885 | VI |
| AAEL005719 | cleavage stimulation factor | D | -0.785 | -0.583 | -0.879 | VI |
| AAEL008716 | conserved hypothetical protein | D | -0.987 |  | -0.876 | VI |
| AAEL011235 | conserved hypothetical protein | D | -0.994 |  | -0.835 | VI |
| AAEL008719 | Sm protein G, putative | D | -1.104 | -0.438 | -0.821 | VI |
| AAEL004960 | hypothetical protein | U | -0.824 | -0.586 | -0.811 | VI |
| AAEL003088 | hypothetical protein | U | -1.215 | -0.751 | -0.802 | VI |
| AAEL009149 | kinectin, putative | D | -0.811 | -0.674 | -0.802 | VI |
| AAEL014483 | conserved hypothetical protein | CS | -0.85 |  | -0.791 | VI |
| AAEL008605 | inosine triphosphate pyrophosphatase (itpase) (inosine triphosphatase) | D | -0.987 | -0.765 | -0.777 | VI |
| AAEL013338 | lethal(2)essential for life protein, l2efl | D | -0.691 | -0.901 | -2.947 | VI |
| AAEL013725 | conserved hypothetical protein | U |  | -1.272 | -2.011 | VI |
| AAEL011423 | sugar transporter | TRP | -0.766 | -1.093 | -1.669 | VI |
| AAEL007045 | conserved hypothetical protein | U | -0.721 | -1.018 | -1.481 | VI |
| AAEL006340 | conserved hypothetical protein | CS | -0.63 | -1.035 | -1.343 | VI |
| AAEL011606 | conserved hypothetical protein | D | -0.752 | -1.165 | -1.323 | VI |
| AAEL010770 | hypothetical protein | U |  | -0.869 | -1.302 | VI |
| AAEL013988 | conserved hypothetical protein | U |  | -0.847 | -1.286 | VI |
| AAEL007946 | glutathione-s-transferase theta, gst | RSM | -0.7 | -0.907 | -1.266 | VI |
| AAEL000065 | conserved hypothetical protein | D | -0.736 | -0.988 | -1.19 | VI |
| AAEL005113 | alpha-esterase | RSM |  | -0.84 | -0.939 | VI |
| AAEL012207 | myosin light chain 1, putative | CS | -0.541 | -0.975 | -0.906 | VI |
| AAEL002431 | conserved hypothetical protein | U | -0.605 | -0.857 | -0.811 | VI |
| AAEL004868 | hemomucin | I |  | -0.811 | -0.807 | VI |
